# Deformation-Induced Phase Transitions in iPP Polymorphs

**DOI:** 10.3390/polym9100547

**Published:** 2017-10-24

**Authors:** Harm J. M. Caelers, Enrico M. Troisi, Leon E. Govaert, Gerrit W. M. Peters

**Affiliations:** 1 Department of Mechanical Engineering, Materials Technology Institute, Eindhoven University of Technology, P.O. Box 513, 5600 MB Eindhoven, The Netherlands; Harm.Caelers@SABIC.com (H.J.M.C.); Enrico.Troisi@SABIC.com (E.M.T.); l.e.govaert@tue.nl (L.E.G.); 2 Faculty of Engineering Technology, University of Twente, P.O. Box 217, 7500 AE Enschede, The Netherlands

**Keywords:** polymorphism, isotactic polypropylene, deformation, phase transitions, uniaxial compression, uniaxial tensile deformation, temperature, in situ X-ray, cavitation

## Abstract

This detailed study reveals the relation between structural evolution and the mechanical response of *α*-, *β*- and *γ*-iPP. Uni-axial compression experiments, combined with in situ WAXD measurements, allowed for the identification of the evolution phenomena in terms of phase composition. Tensile experiments in combination with SAXS revealed orientation and voiding phenomena, as well as structural evolution in the thickness of the lamellae and amorphous layers. On the level of the crystallographic unit cell, the WAXD experiments provided insight into the early stages of deformation. Moreover, transitions in the crystal phases taking place in the larger deformation range and the orientation of crystal planes were monitored. At all stretching temperatures, the crystallinity decreases upon deformation, and depending on the temperature, different new structures are formed. Stretching at low temperatures leads to crystal destruction and the formation of the oriented mesophase, independent of the initial polymorph. At high temperatures, above $T\alpha_{c}$, all polymorphs transform into oriented *α*-iPP. Small quantities of the initial structures remain present in the material. The compression experiments, where localization phenomena are excluded, show that these transformations take place at similar strains for all polymorphs. For the post yield response, the strain hardening modulus is decisive for the mechanical behavior, as well as for the orientation of lamellae and the evolution of void fraction and dimensions. *β*-iPP shows by far the most intense voiding in the entire experimental temperature range. The macroscopic localization behavior and strain at which the transition from disk-like void shapes, oriented with the normal in tensile direction, into fibrillar structures takes place is directly correlated with the strain hardening modulus.

## 1. Introduction

Isotactic polypropylene (iPP) shows several crystal modifications (polymorphs) [1,2]. Depending on chain architecture [3], additives [4] or the conditions experienced during processing [5] the formation of either one of them can be enhanced. The structures most often found are the monoclinic α-, the pseudo-hexagonal β- [6,7,8], the orthorhombic γ- [2,3,9,10] and the metastable meso-phase [11]. These crystals form a morphology in which the crystalline layers (lamellae) are alternated with amorphous layers. In a recent study, the authors presented remarkable similarities in the yield and failure kinetics of the α-, β- and γ-iPP, obtained from uni-axial compression experiments, even though the crystal structures are very different [12]. For the intrinsic material response (true stress versus true strain), however, some interesting differences were found, mainly in the post-yield behavior. It is well known that the softening and hardening of iPP, displayed in the region after yielding, involves a number of structural modifications among which are crystal destruction/melting, orientation of the surviving crystallites and the amorphous network and recrystallization (possibly in other crystal-phases [13,14,15]). Furthermore, the typical alternating heterogeneous structure of a relatively soft amorphous domain and a stiff crystal part introduces stress and strain concentrations on a local scale [16]. In the case of uni-axial extension, this gives rise to an additional phenomenon. Due to the negative hydrostatic pressure that develops while stretching, cavitation or voiding is initiated when the cavitation strength of the amorphous phase, which is lower than that of the crystals, is exceeded [17].

From an engineering point of view, it is of great importance to understand the relation between the various (micro- and meso-scale) structural features of the material and the behavior observed on a macroscopic scale, and therefore, this has been the subject of several studies. The structural evolution of α- [13,15,18,19,20], β- [21,22,23,24] and γ-iPP [14,21,25] was studied for several loading geometries. In general, cavitation is observed in the case of uni-axial tensile deformation, and it was found to affect yield stress and the properties at large strain tremendously.

Because of this strong coupling, Humbert et.al. investigated the initiation of voiding extensively [26] and concluded that, depending on molecular topology and the micro-structural parameters, cavitation can either proceed or follow the onset of plasticity. They related the type of cavitation, i.e., homogeneous or heterogeneous, to a critical lamellar thickness $l_{cc}$. Below this thickness, the critical shear stress for plastic crystal deformation is exceeded before cavitation takes place, while at higher thickness, the critical stress for cavitation is exceeded before the crystals deform plastically. The effect of lamellar thickness on cavitation found by Wang et al. [27] can be explained with this framework. The influence of temperature was further investigated by Xiong et al. [28], who also modeled the cavitation/shear competition successfully [29].

In all these studies, the amorphous material plays a crucial role, for example because it determines the critical cavitation stress. Starting from that perspective, some studies were devoted to develop techniques to quantify the Young’s modulus of the amorphous phase [30] and the strain hardening modulus from tensile experiments [31]. The Young’s modulus of the amorphous phase in the initial stages of deformation can be determined as shown by Xiong et al. [30], who used a procedure based on in situ SAXS and WAXD tensile experiments. They found a decreasing Young’s modulus of the amorphous network with increasing crystallinity and temperature. With respect to the strain hardening modulus it is concluded that at large strains the role of the network is dominant over the residual crystalline structures, particularly in the case of tensile deformation [32,33]. In this perspective disentanglement induced cavitation at large strains, and void propagation via network relaxation were investigated, as well [19,20], and found to be able to reduce the strain hardening modulus to a large extent. Even in strongly oriented material, whitening as a result of cavitation can still occur. Subsequent to voiding and the structure transformation into a fibrillated system, the amount of tie molecules is decisive for the final strength before fracture/failure takes place, as is shown by Ishikawa et al. [34].

Besides the structural features (nanometer scale) and the loading conditions discussed before, the relation between the crystal unit cell structure itself (Angstrom scale) and the cavitation process is investigated for α- and β-iPP [35]. Using SEM techniques, they showed that α-spherulites show micro-cracking in tensile loading. Cavitation takes place at the early stages of deformation and starts at the spherulite boundaries or in the equatorial region [35]. Contrarily, the β-phase deforms plastically up to high deformations. Cavitation starts in the center of the spherulite and subsequently propagates to the equatorial regions [35]. Basic mechanisms like lamellar separation, plastic slip and crazing seem to be involved. Due to the cross-hatched structures of α-iPP lamellae, an increased stiffness is reported since crystalline domains are considered to form a connected physical network [35,36]. The resistance against plastic flow is significantly less for β-iPP when compared to α-iPP, even at deformation where the junctions of the cross hatched structures are destroyed. Therefore, intrinsic mechanical properties of crystals are more likely to be involved in plastic behavior than their micro-structural arrangement [13,36,37]. Due to reduced chain interactions and lower packing density in β-crystals, an enhanced chain mobility is obtained [36,37]. An important limitation of the SEM observation [35] is that the assessed length scale is relatively large, especially compared to the data generated with X-ray techniques.

Despite the large number of studies in this field, a detailed comparison between α-, β- and γ-iPP, tested in the same conditions, is still missing. The deformation and resulting phase transitions, onset of voiding, lamellar growth or destruction and the orientation are revealed in this work. Moreover, the relation of events taking place at the nano- and micro-scale level with the macroscopically intrinsic mechanical response is still unclear. Therefore, in this study in situ WAXD and SAXS experiments are performed in combination with compression and tensile experiments. The compression experiments allow one to investigate the phase transitions as a function of true stress and strain. Cavitation does not occur in compression tests due to a positive hydrostatic pressure. Due to the experimental restrictions, related to the compression setup blocking the diffracted X-ray, the maximal achievable compressive strain is restricted to a true strain of about 80%. The tensile experiments allow one to deform the material up to large strains and investigate voiding. The disadvantage is that it is difficult to relate the phase transitions to true strain. The combination of the two mechanical tests, together with a comparison of the different polymorphs deformed at the exact same conditions, offers a complete overall picture.

This work provides insight on the sequence in which all these events, at the level of crystal structure (Å-scale), lamellae (nm-scale) and voids (µm-scale), take place as a function of deformation and temperature. It deals with the structural changes occurring in uni-axial compression and extension of α-, β- and γ-iPP. This detailed picture of what happens upon deformation allows us to explain the material behavior on a macroscopic scale in terms of micro-scale events. Since the amount of generated data is rather large, a small overview of how the results are presented is given first:

- First, the mechanical response obtained from the tensile experiments is shown, together with the calculated true stress as a function of the true strain. The behavior is discussed and compared with the intrinsic material response measured in uni-axial compression.
- The structural evolution in terms of phase transitions, crystal plane orientation and slip is revealed by WAXD experiments combined with uni-axial tensile tests. The crystal phase transitions are measured in compression experiments, as well, allowing one to compare these results with the ones obtained from tensile experiments (phase transitions) and couple them to the true strain.
- The combination of SAXS experiments with uni-axial tensile tests provided insights on the onset of voiding, lamellar orientation and evolution of amorphous and lamellar thickness. Furthermore, a transition of initially disk-like average void structure to a fibrillar structure was found.

## 2. Materials and Methods

### 2.1. Material and Sample Preparation

**Figure 1:**
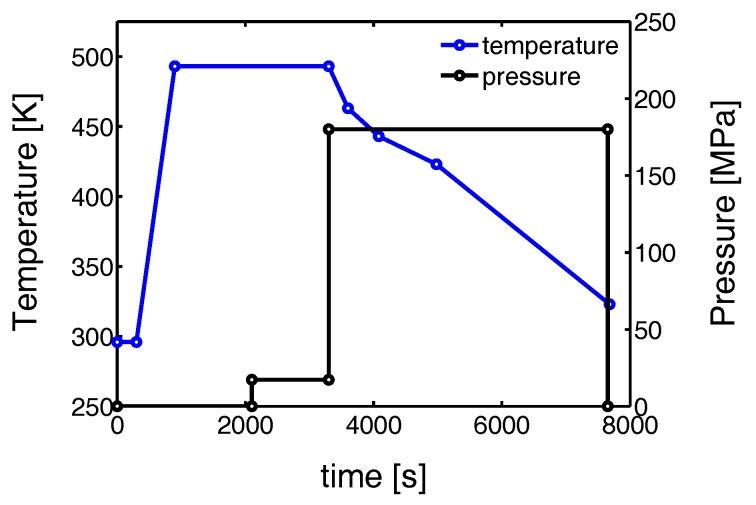
Schematic of the pressure-temperature protocol, used to prepare γ-iPP samples.

In this work, an isotactic polypropylene homo polymer was used (Sabic) with a weight averaged molar mass *M*w of 320 kg/mol and a polydispersity index *M*w/*M*n of 5.4. Sheets of iPP material with a thickness of approximately 1 mm, containing monoclinic α-crystals, were prepared by compression molding. The granular material was placed in a mold and heated to 230 °C, after which a pressure of about 5 MPa was applied stepwise. The material was kept under these conditions for 10 min to erase the thermo-mechanical history. Then, the solidification was induced by putting the mold in a cold press (25 °C) for three minutes. The preparation of sheet material containing pseudo-hexagonal β-crystals was done following the same procedure but with a material containing 0.1 wt % β-specific nucleating agent (NJSTAR NU100, New Japan Chemical Group, Osaka, Japan). To obtain the highest possible β-phase content, solidification was induced by cooling for 3 min in a press set at a temperature of 90 °C, after which the sample was cooled further in ambient conditions to room temperature. For the preparation of samples containing γ-crystals with an orthorhombic unit cell structure, a custom build hydrostatic compression tool is used [12]. The temperature and pressure history, applied during processing, is given in Figure 1. The preparation procedure is based on the work of Mezghani et al. [10]. First the temperature is increased to melt the iPP granulate. After erasing the thermo-mechanical history and the remaining air, a pressure of 180 MPa is applied. Subsequently the sample is slowly cooled to 150 °C, which is sufficiently low to crystallize (The melting temperature increases approximately 30 °C for a pressure increase of 100 MPa [38] and thus the undercooling increases with 30 °C). Finally, when the crystallization process is completed, the sample is cooled further by blowing compressed air through the cooling channels. After reaching room temperature cylindrical sheets of approximately 1 mm in thickness were taken out of the mold. All the iPP-sheets were stored at room temperature for two months before tensile experiments were performed. Thick plates of α-, β- and γ-iPP were prepared following the same sample preparation procedure as described in [12].

### 2.2. Mechanical Testing

**Figure 2:**
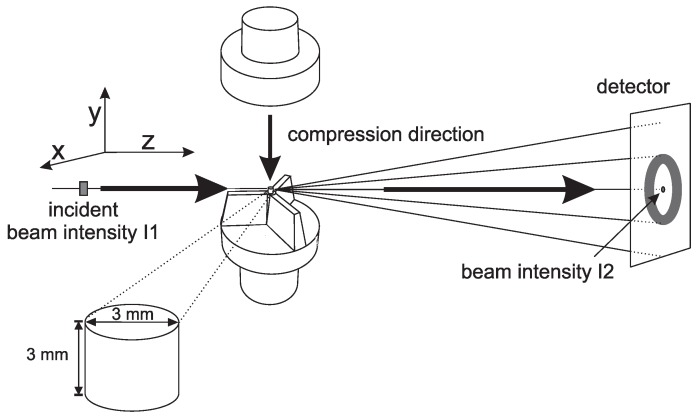
Schematic representation of the compression setup, combined with in situ X-ray experiments.

Two loading geometries were used for mechanical testing procedures, i.e., uni-axial compression and uni-axial tensile. Compression experiments were performed with a well defined constant true strain rate. In this way true stress as a function of the true strain can be related to structural changes. A special tool was developed to prevent the compression setup from blocking the diffracted intensity during in situ X-ray measurements to the highest possible strain; see Figure 2.

**Figure 3:**
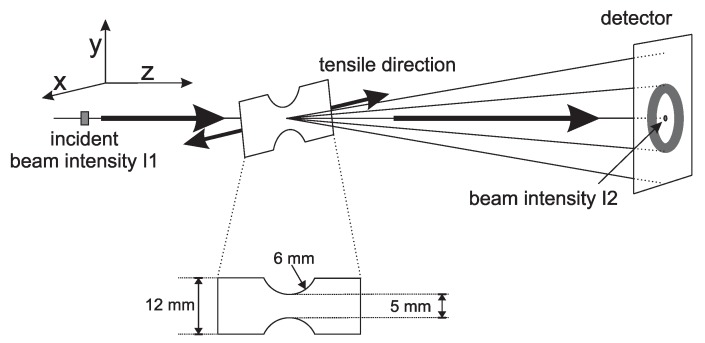
Schematic representation of the tensile setup, combined with in situ X-ray experiments.

The in situmechanical experiments were performed with a Zwick Z5.0 (Ulm, Germany) and the cylindrically-shaped samples, with dimensions of Ø 3 × 3 mm${}^{2}$, were machined from the thick plates. With a custom built oven, temperatures of 110 °C were applied. In the ex situ compression experiments, performed on β- and γ-iPP, samples of Ø 6 × 6 mm${}^{2}$ were used. Since in uni-axial compression a true strain can be applied at a constant rate, and the true stress is measured, no geometrical affects are expected. After the application of a true strain of about 80%, the experimental setup starts to block the diffracted or scattered intensity. In order to study structural evolutions at higher strain, tensile experiments were performed. In this way, structural changes, even the ones taking place far beyond yielding, could be monitored. Furthermore, the onset and the evolution of the shape and dimensions of voids can be measured during tensile deformation. A disadvantage is that it is not possible to control the true strain rate. In tensile deformation the material deforms inhomogeneously after reaching the yield point. To force the tensile sample to neck in front of the beam, dumbbell shaped tensile bars were cut from the iPP-sheets, using a punch according to Figure 3. Another important drawback is that the local strain of the material in the beam area, related to the observed patterns, cannot be determined directly. However, an alternative approach will be explained in Section 2.3.4. A Linkam TST 350 tensile stage (Tadworth, UK) is used to stretch the samples. Temperatures were chosen at 25, 50, 80 and 110 °C respectively, while a stretching speed of 12 μm/s was applied, being equivalent to an engineering strain rate of $8\times 10^{-4}\;s^{-1}$. The samples were stretched in horizontal direction, schematically illustrated in Figure 3. In this schematic, the incident beam intensity $I_{1}$ and the intensity measured with the photo diode $I_{2}$ are also indicated.

Stretching the γ-iPP samples as prepared, resulted in brittle failure at temperatures below the α-transition. Obviously, this is not desired from an experimental point of view. Therefore, the ductility of the γ-iPP samples is improved with a thermal rejuvenation treatment just before the tensile experiment. This turned out to be a successful method to obtain the formation and growth of a stable neck. By keeping the sample for 300 s at a temperature of 85 °C, the constrained amorphous domains in the vicinity of the crystals were rejuvenated, after which the samples were quenched to room temperature. The drop in the yield stress resulting from this treatment is sufficient to obtain ductile behavior [12,39,40].

### 2.3. X-ray Techniques and Analysis

X-ray experiments were carried out at the Dutch-Belgian beamline BM26 (DUBBLE) in the European Synchrotron and Radiation Facility in Grenoble, France [41]. To monitor the structural evolution upon stretching at the level of the unit cell, lamellae and voids, the same set of experiments was repeated three times:

- The 2D WAXD patterns where recorded with a Frelon detector. This detector has a pixel size of 97.65 × 97.65 µm${}^{2}$ and was placed at approximately 140 mm from the sample. The acquisition time was 2.5 s.
- The 2D SAXS patterns where recorded with a Pilatus 1 M detector. Simultaneously with these patterns, a Pilatus 3 K was used to record WAXD patterns. From now on, we will refer to these patterns as 1D WAXD-patterns. The pixel size of the Pilatus 1 M and 1 K was 172 × 172 µm${}^{2}$, with a sample to detector distance of approximately 2707 mm (for SAXS) and 283 mm (for WAXD). At this distance the Porod region is included in the SAXS data. Again the acquisition time was 2.5 s.
- Another set of 2D SAXS experiments were performed with a sample to detector distance of approximately 7258 mm, allowing one to obtain scattering data at low *q*-values. Again these frames where acquired simultaneously with 1D WAXD patterns, recorded with the Pilatus 3 K placed at a distance of approximately 183 mm, and an acquisition time of 2.5 s.
- During the compression experiments, in situ WAXD patterns where recorded using a Frelon detector. This detector is the same as the one used for the 2D WAXD experiments in tensile deformation, but was now placed at approximately 200 mm from the sample. In this case the acquisition time was 1 s.

The wavelength in all the experiments was λ=1.033Å. The size of the beamspot was approximately 0.3 × 1 mm${}^{2}$. The incident beam intensity $I_{1}$ is measured using an ionization chamber and the transmitted beam intensity $I_{2}$ was measured with a photo diode

#### 2.3.1. Data Reduction

To extract results from the X-ray experiments, the data have to be normalized and corrected. In the case of the WAXD experiments a Frelon detector was used and as a result dark current $I_{dc}$, i.e., the signal recorded with a closed shutter, has to be subtracted. This is done on both the patterns recorded with ($I_{m}$) and without the sample ($I_{bkg}$). This correction is not needed for the patterns taken with a Pilatus detector because of the absence of readout noise and dark current. To correct for the air scattering the background $I_{bkg}$ is subtracted. For proper subtraction $I_{bkg}$ was first corrected for the ratio $I_{1}/I_{1,bkg}$ between the incident beam intensity and the background. Next, a correction factor $C=I_{1,bkg}/I_{2,bkg}$ is introduced to compensate for the fact that different devices were used to detect the incident beam intensity $I_{1}$ and the intensity recorded by the photo diode in the beam stop $I_{2}$. The correction for the attenuation due to the presence of a sample is then $C\cdot I_{2}/I_{1}$. In the case of tensile experiments the sample thickness $d_{t}$ decreases during the experiment and the transmission *T* is not constant. To correct for these contributions, Equations (1) and (2) have to be used,

(1) $$d_{t}=\mu \cdot ln\left(I_{1,t}/\left(C\cdot I_{2,t}\right)\right)$$

(2) $$T=\frac{C\cdot I_{2}}{I_{1}}$$

where μ is the absorption coefficient of the sample that can be determined from the intensity measured for the initial sample thickness $d_{0}$. Combining all the corrections results in [42]:

(3) $$I_{cor}=\frac{\frac{\left(I_{m}-I_{dc}\right)}{I_{1}}-C\cdot \frac{I_{2}}{I_{1}}\frac{I_{1}}{I_{1,bkg}}\frac{\left(I_{bkg}-I_{dc}\right)}{I_{1,bkg}}}{T\cdot d_{t}}$$

#### 2.3.2. Phase Composition

**Figure 4:**
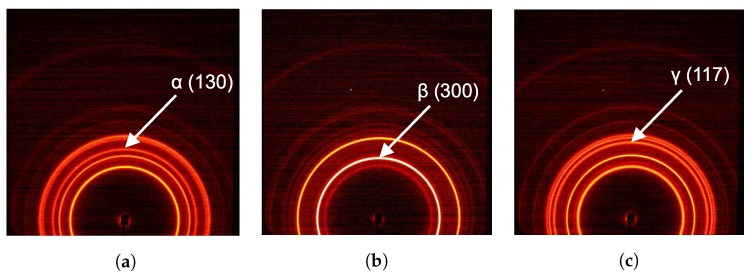
2D WAXD patterns of iPP, characteristic for (**a**) α-iPP; (**b**) β-iPP and (**c**) γ-iPP, as prepared for this study . The diffraction peak unique for α-iPP is the third clear one going from the center towards outside of the pattern. The same holds for γ-iPP, whereas the diffraction ring specific for β-iPP is the most clear one in the β pattern. All characteristic peaks are indicated in the figure.

The crystallographic structures of iPP display a unique diffraction pattern, with intense scattering at angles that are related to specific crystal plane d-spacings by Bragg’s law. In Figure 4 typical 2D diffraction patterns of isotropic α-, β- and γ-iPP are shown.

The most clear diffractions of α-iPP come from the crystal planes corresponding to d-spacings of 6.26Å (110), 5.24Å (040) 4.78Å (130) and 4.17Å/4.05Å (111)/(041). For the γ-iPP the 2D-pattern looks similar and the biggest difference can be observed from the third diffraction ring. The crystal planes and corresponding d-spacings are 6.39Å (111), 5.20Å (008) 4.38Å (117) and 4.17Å/4.05Å (202)/(026). For β-iPP only two important diffraction peaks can be observed at d-spacings of 5.50Å (300), 4.19Å (301) respectively. The arrows in Figure 4 indicate characteristic reflections of α-iPP (Figure 4a), β-iPP (Figure 4b) and γ-iPP (Figure 4c). When these structures orient, the intensity migrates azimuthally to specific angles where the diffracted intensity concentrates.

To obtain quantitative information about the phase composition upon stretching, the patterns are radially integrated over an azimuthal angle of 180${}^{\circ }$. Voigt functions were fitted to the integrated 1D patterns to quantify crystal fractions as a function of strain and temperature. As a first step of the automated peak fitting procedure, the amorphous halo was fitted. After subtraction of the halo, Voigt functions were fitted on the first frame of the series. Here, the sample consisted of almost solely α-, β- or γ-iPP. Upon deformation the diffraction peaks can move to slightly higher or lower *q*-values. Therefore, the initial peak positions were not fully constrained, and allowed to move over a range of ± 0.25 nm${}^{-1}$. Peaks that are present at the initial peak positions (± 0.25 nm${}^{-1}$), were then subtracted from the intensity pattern. After sufficient deformation, the residual signal can contain additional peaks, resulting from the phase transitions. The newly formed peaks, that are subsequently fitted, represent the characteristic diffraction of other crystal phases. Note that these are the only reflections that can be clearly distinguished. The mesophase is defined as everything that is left after the subtraction of the amorphous phase and all the crystal diffractions, and was also fitted with Voigt functions. Finally, after fitting all the peaks of an integrated WAXD pattern, an additional optimization routine was used to fit the exact height and position of the crystal diffraction peaks. In this optimization run, the shape of the peaks was constrained to the values determined in the first peak fitting routine. The expected error that is made for this kind of fitting procedures can in general easily be in the order of 5% to 10%. Within this study, the data are treated the same way every time, and the fitting is done according to a consistent procedure. When comparing between the different results, this consistency is believed to be the most important feature for reducing the relative errors, made in the determination of the phase composition.

**Figure 5:**
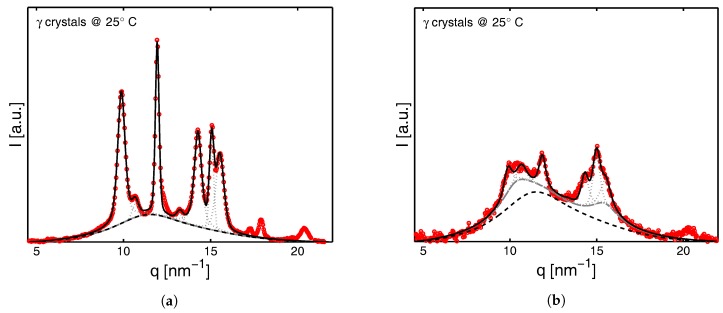
(**a**) An example of a deconvoluted undeformed γ-iPP sample and (**b**) a deformed γ-iPP sample, stretched at 25 °C. The dashed black line is the amorphous halo, the dotted gray lines are the peak fittings, the solid gray line represents the mesophase and the solid black line is the sum of the fitted peaks. The red markers represent the radially integrated pattern obtained from the experiments.

In Figure 5a,b two typical examples of a deconvoluted signal of γ-iPP are shown. The first one, Figure 5a, is the sample at room temperature prior to deformation and the second one, Figure 5b, is the sample after deformation (last image in top row of Figure 15).

The dashed black line in this figure is the amorphous halo that was fitted to the pattern to determine the weight fraction of the crystallinity according to:

(4) $$\chi_{w}=\frac{A_{tot}-A_{a}}{A_{tot}}$$

where $A_{tot}$ is the total diffracted intensity (integrated area) and $A_{a}$ is the integrated area of the scaled amorphous halo (determined on quenched low tacticity iPP with negligible crystallinity). The dotted gray lines represent Voigt functions that were fitted on the diffraction peaks and used to determine the weight fraction of α-, β-, γ- or meso-iPP according to:

(5) $$\chi_{i}=\chi_{w}\cdot \left(\frac{A_{i}}{A_{\alpha }+A_{\beta }+A_{\gamma }+A_{meso}}\right)$$

where $A_{\alpha }$, $A_{\beta }$ and $A_{\gamma }$ are the surfaces corresponding to the characteristic α-,β- and γ-reflections of the (110), (300) and (111) planes respectively. $A_{meso}$ refers to the area of the two mesophase peaks. In Figure 5, the gray lines correspond to this latter phase and, together with the amorphous part (black dashed lines) and the crystal reflections (dotted gray lines), the total (black line) fits the measured patterns very well. The weight fraction of α-, β- and γ-iPP, as well as the crystallinity $\chi_{w}$, is a mass percentage because the diffraction is proportional to the number of diffracting centers, i.e., the number of crystals, and hence the mass.

#### 2.3.3. Determination of lp, lc and la

Two different methods are used to obtain the long period, the lamellar thickness and the thickness of the amorphous layer. The first one is based on Bragg’s law and follows from the integrated Lorentz corrected scattering (SAXS) intensity, given by:

(6) $$I_{1}\left(q\right)=I\left(q\right)q^{2}$$

where *I* is the intensity and q is the scattering vector. The long period then follows via:

(7) $$l_{p}=\frac{2\pi }{q_{I_{1},max}}$$

with $q_{I_{1},max}$ the maximum of the Lorentz corrected intensity. Since the long period is constructed of an amorphous volume and a crystalline volume , it is straightforward that the lamellar thickness $l_{c}$ follows from:

(8) $$l_{c}=\frac{2\pi }{q_{I_{1},max}}\chi$$

where χ is the volume percentage of the crystallinity. This percentage is obtained from WAXD experiments via [32] :

(9) $$\chi =\frac{\frac{\chi_{w}}{\rho_{c}}}{\frac{\chi_{w}}{\rho_{c}}+\frac{1-\chi_{w}}{\rho_{a}}}$$

In this equation the mass fraction of crystals, $\chi_{w}$, follows from Equation (4). The amorphous layer thickness can then easily be obtained by subtraction of the lamellar thickness from the long period:

(10) $$l_{a}=l_{p}-l_{c}$$

An alternative way to determine these quantities is via the 1D auto-correlation function $\gamma_{1}\left(r\right)$ [43,44]. For spherical symmetry this can be calculated with:

(11) $$\gamma_{1}\left(r\right)=\frac{1}{Q}\int_{q_{0}}^{q_{\infty }}I_{1}\left(q\right)cos\left(qr\right)\;dq,$$

where *r* is the real space and *Q* is the scattering invariant defined by:

(12) $$Q=\int_{q_{0}}^{q_{\infty }}I_{1}\left(q\right)\;dq,$$

To extrapolate the experimentally assessed range of the scattering vector, Debye-Bueche [45] and the Porod law [46] are used, respectively. The long period, amorphous layer thickness and lamellar thickness were then determined as described by Stein et al. [47]. The most important difference between the determination of these quantities via Bragg’s law and the auto-correlation function is that the latter one gives higher values for the lamellar thickness, and thus lower values for the amorphous domain thickness. This originates from the interface between the two phases which is more dense than the bulk amorphous parts and therefore considered to be part of the lamellae in the approach using the autocorrelation function.

**Figure 6:**
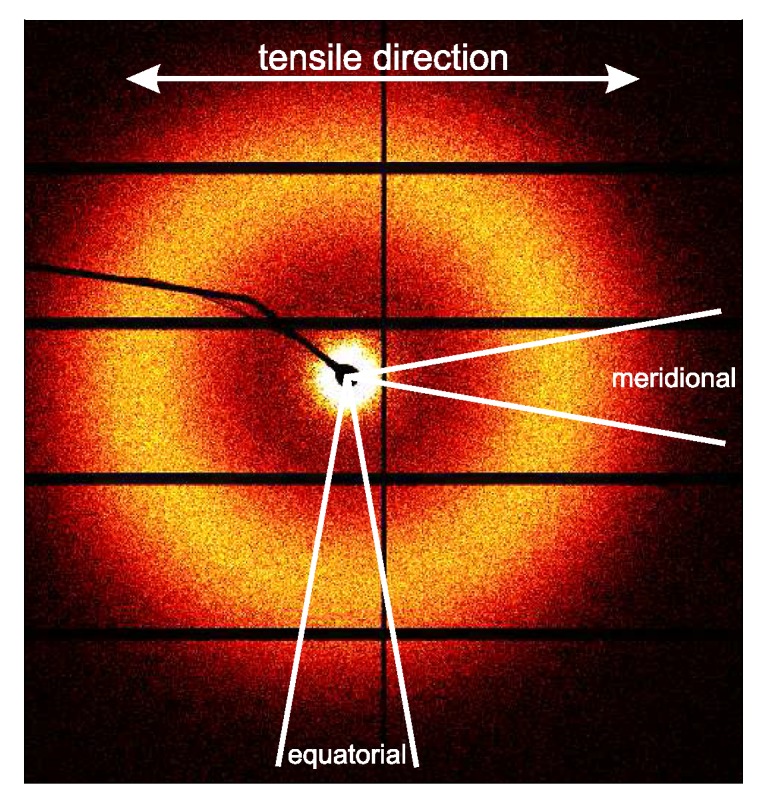
Regions used for radial integration of the SAXS patterns.

Lamellar quantities like the long period $l_{p}$ can be determined from the scattering in either the meridional region, or the equatorial region, parallel and perpendicular to the tensile direction respectively (see Figure 6).

This allows one to distinct between these features in either the tensile direction (integration of the meridional region) or the transverse direction (integration of the equatorial region). Obviously, this is only of interest at relatively low strains before lamellae start to break, voiding takes place or, depending on the temperature, the material recrystallizes.

#### 2.3.4. Determination of the Strain

Due to the shape of the tensile samples and the inhomogeneous deformation after yielding, the true strain of the sample volume in front of the X-ray beam cannot be calculated directly from the applied engineering strain. However, the X-ray data can also be used to determine the draw ratio of the sample volume in front of the beam [48]. This allows us to obtain all structural parameters as a function of actual deformation. It should be noticed that, although we intended to create an uni-axial deformation, this was not guaranteed due to the initial sample shape, which was forced by the experimental conditions (i.e., the start of a neck in a known position where the beam is positioned), and the complex plastic deformation mechanisms accompanied with localization phenomena and crystal phase transitions. Moreover, the deformation mode (anything between uni-axial and planar) can change during the deformation path and these changes can be temperature, deformation rate, void formation and phase dependent. To deal with this complex situation we will analyze our experimental data by considering two limiting cases: pure uni-axial and pure planar deformation.

To determine the true strain, the draw ratio $\lambda_{l}$ in the tensile direction, defined as:

(13) $$\lambda_{l}=\frac{l_{t}}{l_{0}}$$

should be calculated first. Here, $l_{0}$ is the initial length of an arbitrary volume in front of the beam at t=0, and $l_{t}$ is the length at time *t*. If we now assume incompressibility, we get:

(14) $$l_{0}w_{0}d_{0}=l_{t}w_{t}d_{t}$$

Combining this with Equation (13), and with the assumption of uni-axial deformation, i.e., the contraction in the tensile-, width- and thickness direction is equal, the width at t=0 ($w_{0}$) and at time *t* ($w_{t}$) can be substituted by the thickness $d_{0}$ and dt respectively, and Equation (15) can then be applied to obtain the actual draw ratio. This calculated ratio is resulting from the decrease of polymeric material in the beam area. Throughout the entire stretching process and the associated deformation of the test specimen, the sample remains larger than the X-ray beam. The latter one has dimensions of 0.3 × 1 mm${}^{2}$, which is clearly smaller than the final sample dimensions.

(15) $$\lambda_{l,uni}=\frac{1}{\lambda_{d}^{2}}={\left(\frac{d_{0}}{d_{t}}\right)}^{2}$$

The sample thickness at time *t*, defined as $d_{t}$, can be straightforwardly calculated using Equation (1), and at time t=0 the initial thickness $d_{0}$ follows from [48]:

(16) $$d_{0}=\mu ln\left(I_{1,t=0}/\left(C\cdot I_{2,t=0}\right)\right)$$

Substitution of Equations (1) and (16) into Equation (15) allows us to determine the draw ratio on the local level of the beam spot, without determination of μ. This ratio is determined on a homogenized volume since the beam dimensions are much larger than the spherulites and the lamellae. To calculate the draw ratio for plane strain conditions, Equation (17) is used.

(17) $$\lambda_{l,pl}=\frac{1}{\lambda_{d}}=\left(\frac{d_{0}}{d_{t}}\right)$$

In this case, the draw ratio in tensile direction equals the draw ratio in thickness direction, but the sample width remains constant upon stretching. The true strain and true stress then follow via:

(18) $$\epsilon_{true,n}=ln\left(\lambda_{l,n}\right)$$

and:

(19) $$\sigma_{true,n}=\sigma_{e}\cdot \lambda_{l,n}$$

for both the assumptions of uni-axial deformation and plane strain conditions. In the latter equation, $\sigma_{e}$ is the engineering stress. The subscript *n* in this equation denotes either uni-axial deformation or plane strain conditions.

**Figure 7:**
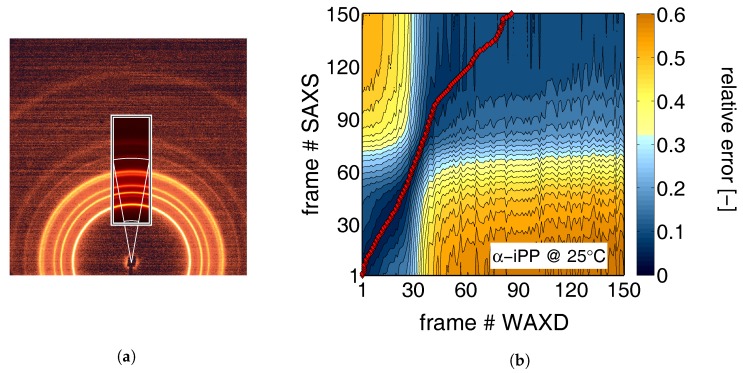
(**a**) Example of a WAXD pattern measured with the Frelon detector. In the inset the WAXD pattern measured simultaneously with SAXS is put into the 2D WAXD pattern; (**b**) An example of a figure used to find the minimal difference between the two WAXD patterns. Numbers on the axes indicate the frame number.

The approach presented here, which is based on the intensity drop as a result of the sample presence, can be used if the following assumption holds:

(20) $$I_{1}=I_{trm}+I_{rfl}+I_{abs}+I_{sct}\approx I_{trm}+I_{abs}$$

where $I_{1}$ is the incident beam intensity, $I_{trm}$ is the transmitted intensity which is measured with the photo-diode, $I_{rfl}$ is the reflected intensity, $I_{abs}$ is the absorbed intensity and $I_{sct}$ is the scattered intensity. However, if voids appear, large density differences are introduced and as a result the scattered part of the intensity is no longer negligible with respect to the transmitted part. Since the scattering from the voids takes place under a very small angle (due to the relatively large length scales involved), the intensity measured in the photo diode during the WAXD experiments is the sum of the scattering due to the voids, and the transmission. This means that for the WAXD experiments the transmitted intensity can be determined even if voids appear, and Equations (1) and (16) can be applied. To obtain the strain for the SAXS experiments, where this is not the case, simultaneously recorded WAXD patterns (Pilatus 3 K) were linked to the 2D-WAXD patterns via superposition, indicated with the rectangular section in Figure 7a. The white lines indicate the slice-shaped area used for radial integration. In Figure 7b, an example of the relation of the SAXS frames as a function of the WAXD frames is shown. The color represents the relative difference between the scaled integrated intensity as a function of the scattering vector *q* of the 2D- and the 1D WAXD patterns. The red markers highlight the minimum. With this coupling, determined for all sets of SAXS-, and their corresponding WAXD experiments, the evolution in sample thickness can be obtained.

On a very local level, an estimate for the strain can be deduced from the changing distance between the crystal planes. Upon stretching, the evolving d-spacing can be used, similar to Xiong et al. [30] according to:

(21) $$\epsilon_{d-space}=\frac{d_{hkl}-d_{hkl,0}}{d_{hkl,0}}$$

to express the evolution in terms of a strain. In this equation $d_{hkl}$ is the d-spacing of a specific plane hkl in time, and $d_{hkl,0}$ is the spacing at time t=0.

#### 2.3.5. Void Fraction

Initially the scattering is caused by the crystals and amorphous phase, but upon deformation, voids appear, and the scattering resulting from these voids becomes dominant. This is accompanied by a strong increase in the scattering invariant due to the large difference in the density of the material and a void. For the calculation of the scattering invariant, cylindrical symmetry is assumed in the loading direction. The normalized scattering invariant is then given by [42]:

(22) $$\frac{Q}{Q_{0}}=\frac{\int_{-\infty }^{\infty }\int_{0}^{\infty }I_{cor}\left(q_{x},q_{y}\right)q_{y}dq_{x}dq_{y}}{\int_{-\infty }^{\infty }\int_{0}^{\infty }I_{cor,t=0}\left(q_{x},q_{y}\right)q_{y}dq_{x}dq_{y}}$$

where $q_{y}$ and $q_{x}$ are the scattering vector in vertical and horizontal direction respectively. $Q_{0}$ is the invariant at time t=0, prior to deformation.

Under the assumption that the we start from a situation without cavities, the void fraction can be calculated directly form the invariant according to [42]:

(23) $$\phi_{v}=\left[\frac{Q}{Q_{0}}-1\right]\cdot {\left[\frac{\chi \rho_{c}^{2}+\left(1-\chi \right)\rho_{a}^{2}}{\chi \left(1-\chi \right){\left(\rho_{c}-\rho_{a}\right)}^{2}}-1\right]}^{-1}$$

with $\rho_{c}$ the crystal density, given by:

(24) $$\rho_{c}=\frac{\chi_{\alpha }}{\chi }\cdot \rho_{\alpha }+\frac{\chi_{\beta }}{\chi }\cdot \rho_{\beta }+\frac{\chi_{\gamma }}{\chi }\cdot \rho_{\gamma }+\frac{\chi_{meso}}{\chi }\cdot \rho_{meso}$$

where $\chi_{i}$ is the mass fraction of crystal phase *i*, with density $\rho_{i}$ (*i* ∼ α, β, γ, meso). The values used for the density of the specific crystal phases are given in Table 1, as well as the density of the amorphous phase. The fractions can be obtained from Equation (5).

## 3. Results and Discussion

Several strain and stress definitions are used in this study depending on the type of results presented. The engineering stress $\sigma_{e}$ and strain $\epsilon_{app}$ (also referred to as apparent macroscopic strain) are calculated with the initial gauge length and the initial cross sectional area. These are used when we intend to link structural transitions to the macroscopically observed tensile behavior. When we want to clarify transitions, the true stress $\sigma_{true}$ and strain $\epsilon_{true}$ are sometimes used. To obtain the strain hardening modulus we use $\lambda^{2}-1/\lambda$ as a strain measure [39] and finally, for the strain determined at the local scale of the lamellae, $\epsilon_{l}$ is used.

### 3.1. The Mechanical Response

#### 3.1.1. Tensile Tests (Mechanical)

**Figure 8:**
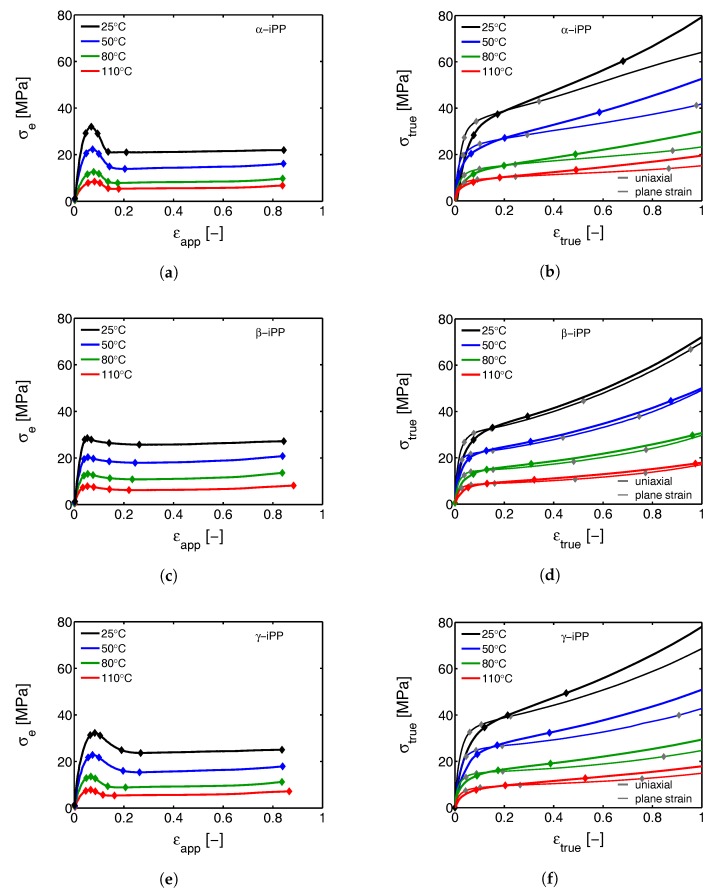
(**a**,**c**,**e**) The engineering stress as a function of the apparent strain for α-, β- and γ-iPP tensile experiments respectively, stretched at different temperatures and a rate of 12 µm/s; (**b**,**d**,**f**) The true stress as a function of true strain, corresponding to figure (**a**,**c**,**e**). The thick solid lines are calculated for the assumption of uni-axial deformation and the thin lines for plane strain. The markers in the figures correspond to the 2D WAXD and SAXS images, shown in the following sections.

In Figure 8a,c,e, the engineering stress as a function of macroscopic apparent strain is shown for tensile tests, performed on different iPP-polymorphs and at multiple temperatures. Initially, linear elastic behavior can be observed, but with increasing strain and stress the deformation becomes plastic, ultimately leading to yield. Temperature facilitates the mobility and, therefore, has a reducing effect on the resistance against yielding. This can also be seen in Table 2, where an overview of the obtained yield stresses at different temperatures is given for α-, β- and γ-iPP.

The two limiting cases of uni-axial extension and plane strain of α-, β- and γ-iPP are shown in Figure 8b,d,f. Up to yielding, where the deformation is close to homogeneous, the discrepancy between uniaxial and planar deformation is mainly observed in the initial elastic stiffness. After yielding a cross-over takes place and, depending on the amount of softening seen in the engineering stress, the uni-axial response increasingly deviates from the plane strain response. For β-iPP, with almost no softening, the difference is rather small.

**Figure 9:**
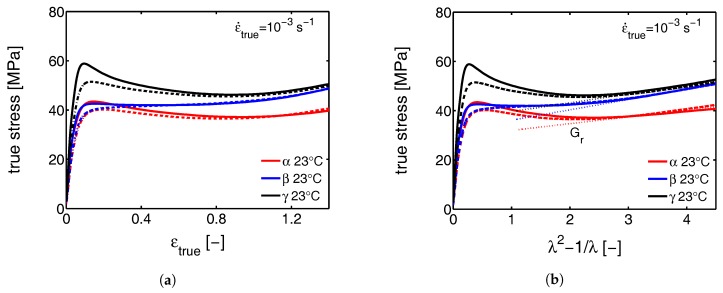
(**a**) True stress-true strain curves for α-, β- and γ-iPP. The results are obtained from uni-axial compression experiments at a strain rate of $10^{-3}\;s^{-1}$ and a temperature of 23 °C on samples with a dimension of Ø6 × 6 mm${}^{2}$ (β- and γ-iPP) or Ø3 × 3 mm${}^{2}$ (α-iPP). The dashed lines are the true stress-true strain response obtained on thermally rejuvenated samples; (**b**) The corresponding Gaussian plots for α-, β- and γ-iPP. The dotted lines represent the strain hardening moduli $G_{r}$.

**Figure 10:**
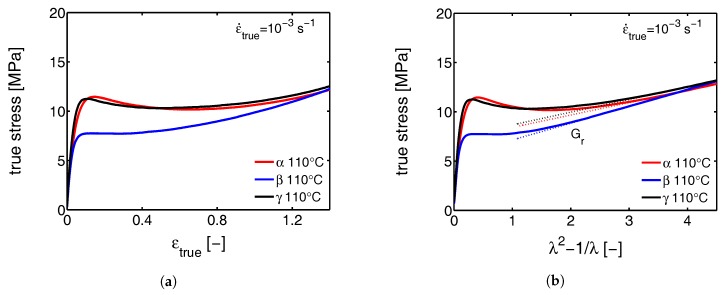
(**a**) The solid lines are the true stress as a function of the true strain obtained from uniaxial compression experiments on α-, β- and γ-iPP, measured at a strain rate of $10^{-3}\;s^{-1}$ and a temperature of 110 °C on samples with a dimension of Ø6 × 6 mm${}^{2}$ (β- and γ-iPP) or Ø3 × 3 mm${}^{2}$ (α-iPP); (**b**) The corresponding Gaussian plots for α-, β- and γ-iPP. The dotted lines represent the strain hardening moduli $G_{r}$.

Although in agreement with the findings presented by G’Sell et al. [49], the true stress-true strain results obtained in the tensile experiments are different from the ones from uni-axial compression, where softening is observed and the yield stress is much higher (see Figure 9a and Figure 10a). First of all, this discrepancy can result from the formation of cavities, that can develop due to negative hydrostatic stresses in the case of the tensile experiments. The cavities appear on a local level and cause macroscopic softening to (partially) disappear [50], even though locally the softening is maintained. This phenomenon is called sequential yielding and can start prior to macroscopic yield, as will be shown in the following sections. Second, the stress and strain fields in a tensile experiment can become highly inhomogeneous after yield, and cause the sample to deform with a variable strain rate. As soon as the sample softens, locally the strain rate strongly increases. This has a reducing effect on the amount of softening observed in the macroscopic mechanical response. Finally, the sample preparation procedure is slightly different for compression and tensile experiments, also having a minor effect. In order to prepare compression samples, slightly thicker plates were compression molded. Because of that, the average cooling rates upon quenching in a cold press are slightly lower. This could lead to small differences in crystallization temperature and lamellar thickness, which manifests itself in the mechanical response in terms of the observed yield stress [51]. Furthermore, extreme differences in cooling rate and crystallization temperature can also have an affect on the strain hardening modulus [32]. In this case, however, the differences in processing are so minor, that it is assumed that it can be neglected.

#### 3.1.2. Compression Tests (Mechanical)

Since the amorphous network is crucial for the mechanical response at high strains [33], the strain hardening modulus $G_{r}$ is determined from compression experiments. From tensile experiments it is not possible to find a qualitative value for the hardening modulus due to the difficulties discussed before. The compression experiments, on the other hand, are perfectly suitable for this goal since localization phenomena are excluded and a well defined constant true strain rate can be applied. Consequently, the intrinsic true stress and true strain can be obtained and the strain hardening modulus can straightforwardly be determined according to Haward [39]. In Figure 9b a Gaussian plot, i.e., the true stress as a function of $\lambda^{2}-1/\lambda$, is shown, for α-, β- and γ-iPP compressed at room temperature with a true strain rate of $10^{-3}\;s^{-1}$. The dashed lines are compression experiments performed under the same loading conditions, but on thermally rejuvenated samples as explained in our previous work [12]. Linear fits, determined in the hardening regime, are represented by the dotted lines. These slopes give an estimation for the strain hardening modulus $G_{r}$. It is clear that β-iPP has the highest strain hardening modulus (4.2 MPa) and α-iPP the lowest (2.2 MPa). The strain hardening modulus of the γ-iPP is in between (3.5 MPa). Moreover, the thermal rejuvenation treatment does not seem to affect the hardening (in this strain regime), but reduces the softening significantly. Good agreement is found when comparing the hardening modulus of α-iPP with the results reported by Schrauwen et al. [32] and Haward [39]. To the knowledge of the authors no research has been devoted to the determination of hardening moduli for β- and γ-iPP.

In our previous work [12] we showed that the contribution of the constrained amorphous phase to the effect of thermal rejuvenation on the yield stress, is almost similar for α- and β-iPP, independent of the strain rate. This thermal rejuvenation treatment has no effect on the hardening modulus, see Figure 9b, but mainly leads to a reduction in the true stress-true strain response at strains around yielding. After yielding the thermally rejuvenated samples coincide again with the aged samples. This observation is in good agreement with the behavior typically observed in aged and, either thermally or mechanically, rejuvenated amorphous polymers [52].

Softening after yielding is a property of an amorphous glassy material. The degree of softening is a direct consequence of the amount of aging a sample has experienced, prior to the mechanical testing. In the case of iPP, at a temperature above the glass transition of the bulk material, and below the melting temperature of the crystals, softening is related to the constrained amorphous phase (which is considered to be in a glassy state). In the vicinity of the relatively immobile crystalline domains, the mobility of the amorphous material is strongly reduced. The extent of the constraints can depend on a number of morphological and crystallographic features. The strength of the secondary interactions in the crystal is related to the density, which is the lowest in case of β-iPP (see Table 1). The lamellar thickness and the density of crystal defects also affect the strength of the constraints. Furthermore, the crossed stacking of chains within the lamellae of γ-iPP is also positively contributing to the constraints. All these constraints are directly coupled to the properties of the crystal. With thermal rejuvenation softening partially disappears. Particularly in γ-iPP a large reduction in yield stress is found, but also in α- and β-iPP a decrease is observed, indicating that the iPP contains constrained amorphous material in all three crystal phases. At 110 °C, where the constrained amorphous material is mobile (above the $\alpha_{c}$-relaxation temperature), both α- and γ-iPP still show some remaining softening, see Figure 10. This means that as a result of deformation, the structural integrity further deteriorates after yielding. As can be observed from Figure 20 in Section 3.2.2, this is not governed by a large reduction in crystallinity. Moreover, this reduction is very similar for the β-iPP. Based on these observations it could be hypothesized that the break-down of the cross hatched crystal network is responsible for the softening in α- and γ-iPP, without destroying the crystals themselves, since these structures are typically present in these polymorphic forms [2,53,54]. In this perspective, the absence of these structures in the case of β-iPP could explain why the mechanical response displays no softening. The trends in the strain hardening moduli at 110 °C are similar to that at 25 °C.

To summarize, from the mechanical behavior in uni-axial compression it is hypothesized that the intrinsic softening observed in polypropylene is an effect, mainly resulting from the constrained amorphous domains in the vicinity of the crystals. However, as follows from the compression experiments performed at 110 °C, combined with the results of the WAXD experiments presented in Section 3.2.2, suggests that the constraints implied by the cross-hatched structures, present in α- and γ-iPP, also contribute to the softening.

### 3.2. WAXD Analysis

#### 3.2.1. Tensile Tests (WAXD)

**Figure 11:**
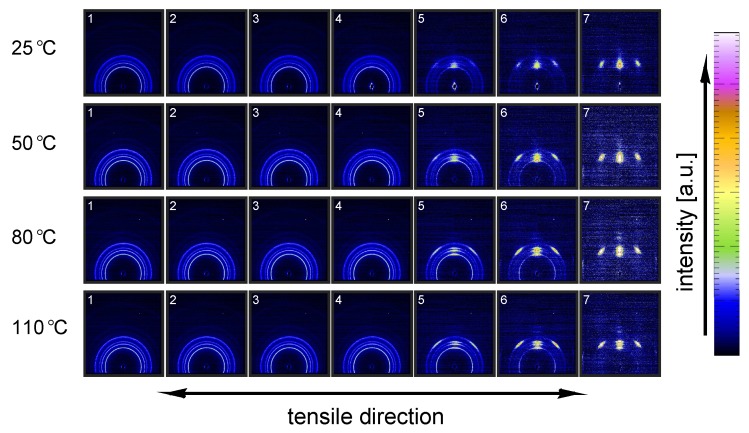
Normalized 2D WAXD patterns of α-iPP stretched at temperatures of 25, 50, 80 and 110 °C from top to bottom. The true strains, determined with the assumption of fully uni-axial deformation, are given as well. The macroscopic strains at which the patterns were taken are indicated by the markers in Figure 8. The stretching direction is horizontal.

Figure 11 shows the evolution of the α-iPP crystal structure during tensile testing in terms of the normalized 2D WAXD patterns, as a function of strain for 4 different temperatures. Since most of the structural changes take place in the macroscopically observed softening regime, i.e., in a small apparent strain range, it is chosen to depict the changes as a function of uniaxial true strain for clarity reasons. The patterns depicted here correspond to the markers in Figure 8. Initially, before straining the sample, an isotropic diffraction pattern is observed which clearly shows the monoclinic α-phase reflections. Upon straining the sample, the pattern becomes slightly elliptical, indicating that the d-spacings of the crystals in the polar regions are extended whereas the distances in the equatorial regions reduce. As a result of further straining, the reflections become less pronounced and the scattering intensity migrates to certain angles, indicating (strong) orientation. At high elongation at room temperature a transition from α-iPP to oriented mesophase can be observed [55], whereas at high temperatures the isotropic α-iPP transforms to a strongly oriented α configuration.

**Figure 12:**
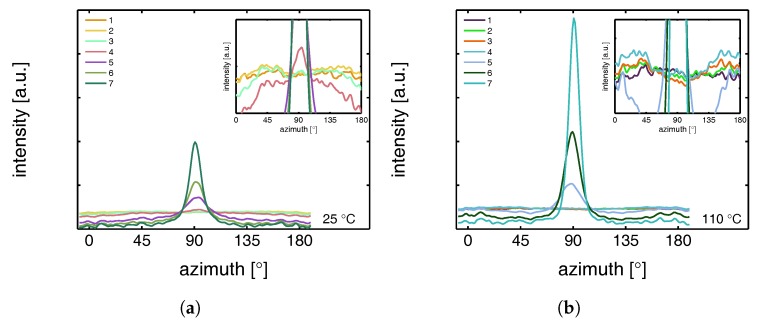
Azimuthal spread of the (110) diffraction of α-iPP at various strains; (**a**) uni-axial stretching at 25 °C and (**b**) 110 °C. The numbers in the legend correspond to the 2D patterns in Figure 11.

This azimuthal orientation is investigated in more detail by integrating the (110) diffraction peak over an angle ranging from 0 to 180°. An angle of 90° represents the equator, while 0 and 180° are the polar regions. This integration was carried out for α-iPP stretched at the lowest and the highest temperature, i.e., corresponding to the top and bottom row in Figure 11, and the results are given in Figure 12. Due to the strong orientation and the high intensity at an azimuth of 90° the minor effects at lower strains are unclear. In the inset an enlarged plot of this region is shown. The intensity transforms from nearly isotropic in the initial stage of deformation to a slightly oriented state prior to yielding.

Straining the sample further leads to selective melting and recrystallization, and orientation of “old crystals” into a strongly oriented mesophase at 25 °C, and α-phase at 110 °C. The peak in the azimuthal integration, representative for these oriented structures shows up first in the yield point and evolves in the softening and hardening regions thereafter. Small intensity maxima at 10° and 170° degrees, observed in the material stretched at 110 °C, indicate the survival or appearance of cross-hatched structures in the stretched sample [56].

**Figure 13:**
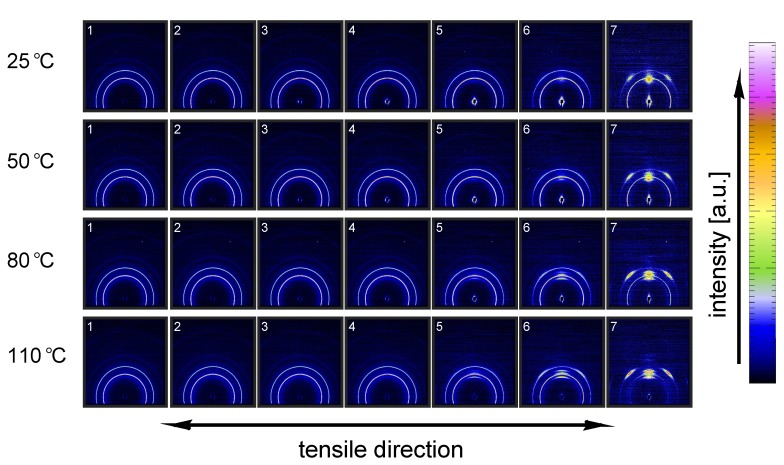
Normalized 2D-patterns of β-iPP stretched at temperatures of 25, 50, 80 and 110 °C from top to bottom. The true strains, determined with the assumption of fully uni-axial deformation, are given as well. The macroscopic strains at which the patterns were taken are indicated by the markers in Figure 8. The stretching direction is horizontal.

A similar figure can be made for β-iPP, see Figure 13. In the initial stages of deformation the isotropic diffraction rings resulting from the pseudo-hexagonal β-phase structure become elliptical due to the (elastic) deformation. Contrary to α-iPP at room temperature, the diffraction of the β-iPP crystals present in the initial isotropic crystal structure seems to be partially maintained during stretching. The diffraction of the β-iPP is more intense than the reflections of the α- and γ-iPP. The scaling of all 2D-WAXD images in this work is the same, and as a result, a larger part of the crystals has to be destroyed to get rid of the β-diffraction peaks. Moreover, due to different localization behavior, the uni-axial true strain belonging to β-iPP in front of the beam is lower than for α- and γ-iPP, even if the same amount of macroscopic strain is applied. Consequently, after finishing an experiment that is stopped at the same macroscopic strain for all iPP samples, the actual true strain reached by β-iPP in front of the beam is clearly lower. This makes a direct comparison complicated. The other part of the initial structure that is destroyed forms new oriented structures. At room temperature the diffracted intensity appears at positions typical for oriented mesophase. At elevated temperature and upon deformation, a transition from β-crystals to oriented and more stable α-crystals takes place.

**Figure 14:**
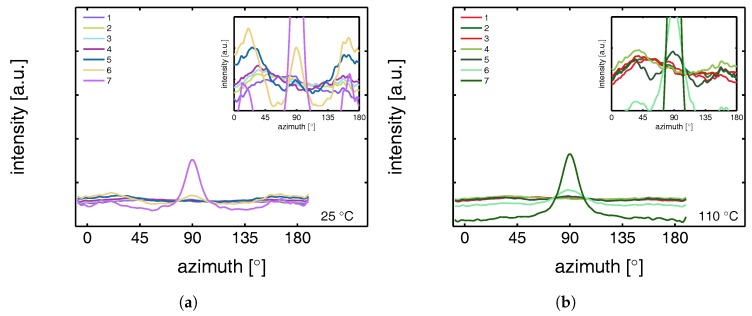
Azimuthal spread of the (300) diffraction of β-iPP at various strains. (**a**) uni-axial stretching at 25 °C and (**b**) 110 °C. The numbers in the legend correspond to the 2D patterns in Figure 13.

From an azimuthal integration over the (300) diffraction peak of β-iPP, deformed at 25 and 110 °C (Figure 14), it becomes clear that the amount of material involved in partial melting and/or orientation is much less (as a function of apparent macroscopic strain) as compared to α-iPP. Only in the last few frames a clear decrease in the intensity at an azimuth of 0 and 180° is observed. The deformation of the initial structure to preferred orientations on the other hand, continues also after the yield point (frame 3). At room temperature, as well as at 110 °C, the first evidence of recrystallization in a new oriented phase is found in frame 5, corresponding to deformation well beyond the macroscopic yield point.

**Figure 15:**
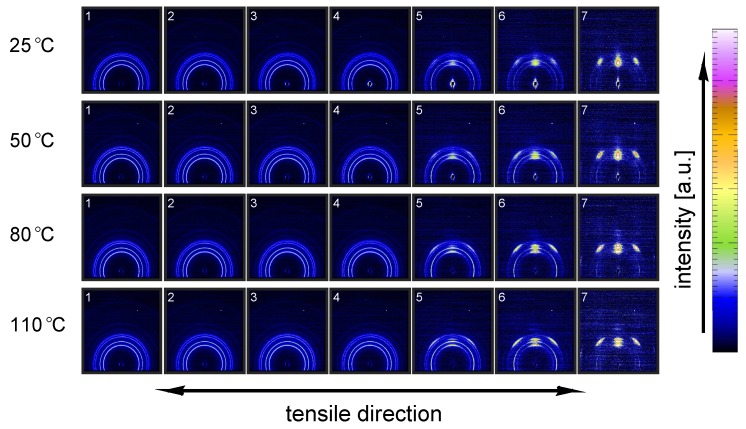
Normalized 2D-patterns of γ-iPP stretched at temperatures of 25, 50, 80 and 110 °C from top to bottom. The true strains, determined with the assumption of fully uni-axial deformation, are given as well. The macroscopic strains at which the patterns were taken are indicated by the markers in Figure 8. The stretching direction is horizontal.

The reflections of the orthorhombic γ-phase become very vague upon deformation at 25 °C, and the scattering transforms into a pattern indicative for oriented mesophase, see Figure 15. Stretching at 50 °C result is a similar response, however, besides mesophase some small amounts of oriented α-iPP seems to be formed. Elongation at 80 and 100 °C results in a transition of isotropic γ-iPP into strongly oriented α-iPP. All these transitions take place after yielding. These 2D-patterns provide qualitative information about the orientation resulting from the stretching.

**Figure 16:**
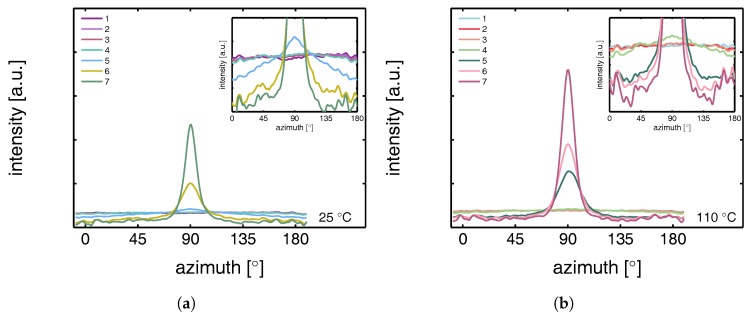
Azimuthal spread of the (111) diffraction of γ-iPP at various strains. (**a**) uni-axial stretching at 25 °C and (**b**) 110 °C. The numbers in the legend correspond to the 2D patterns in Figure 15.

The azimuthal scan of the (111) peak and the evolution as a result of the deformation is given in Figure 16, for the γ-samples deformed at 25 and 110 °C. Interestingly, the intensity remains rather isotropic until the onset of the softening after yielding (frame 5–7). The absence of the off-axis orientation means that the γ-crystals do not allow for partial orientation of the initial crystallites. After yielding, when selective melting and recrystallization takes place, the intensity of the (111) peak in the polar regions starts to decrease and the newly formed structures appear at an azimuth of 90°.

**Figure 17:**
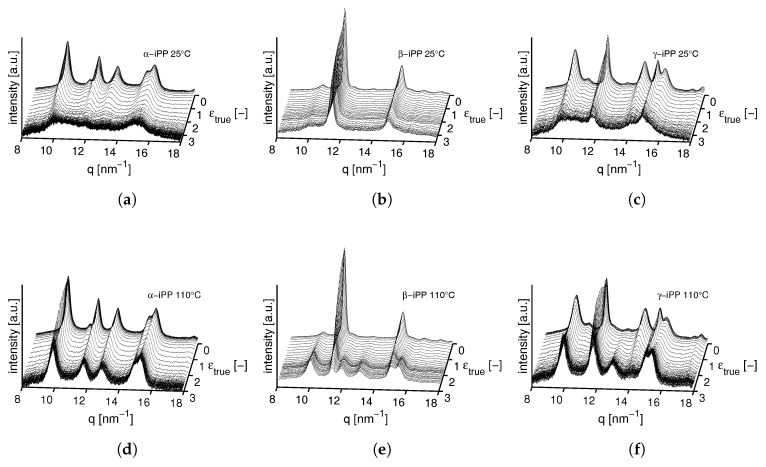
The integrated intensity as a function of the scattering vector *q*. From (**a**–**c**) we see the evolution of the crystallinity upon stretching at 25 °C of the α-, β- and γ-iPP respectively. Similar results, obtained from stretching experiments performed at 110 °C, are shown in (**d**–**f**). The structural evolution is given as a function of the true strain.

In Figure 17 the results of radial integration of the patterns measured on all crystal phases, stretched at testing temperatures of 25 and 110 °C are plotted as a function of the scattering vector *q* and the true strain $\epsilon_{true}$. At 25 °C the reflections of α- and γ-iPP become less intense and broaden, particularly in the macroscopic softening region, corresponding to true strains of about 0.2 to 2 [-]. The β-crystals, on the other hand, seem to maintain their crystallographic structure, and the biggest change observed is broadening of the peaks. At 25 °C and high strain, α- and γ-iPP eventually transform into a 1D intensity typical for the mesophase. In the figures corresponding to elongation at 110 °C it is evident that the α-reflection seems to be maintained rather well, whereas for β and γ-iPP the characteristic reflections disappear, simultaneously with the appearance of the α-reflection. The reflections remain sharp with well defined peaks up to high true strains.

**Figure 18:**
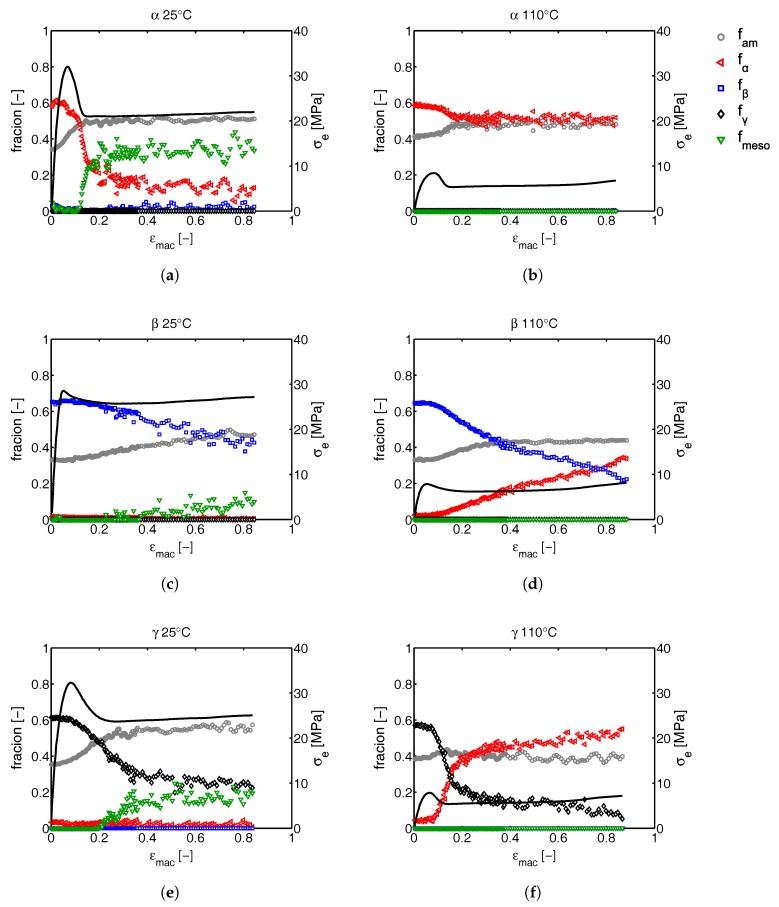
The crystal phase fractions obtained from the WAXD experiments. In (**a**,**c**,**e**) we see the evolution of the crystallinity and phase fractions upon stretching at 25 °C. Figure (**b**,**d**,**f**) show the result of the tensile experiments performed at 110 °C. From top to bottom we see α-, β- and γ-iPP. The solid lines are the macroscopic engineering stress as a function of the apparent macroscopic strain.

**Figure A1:**
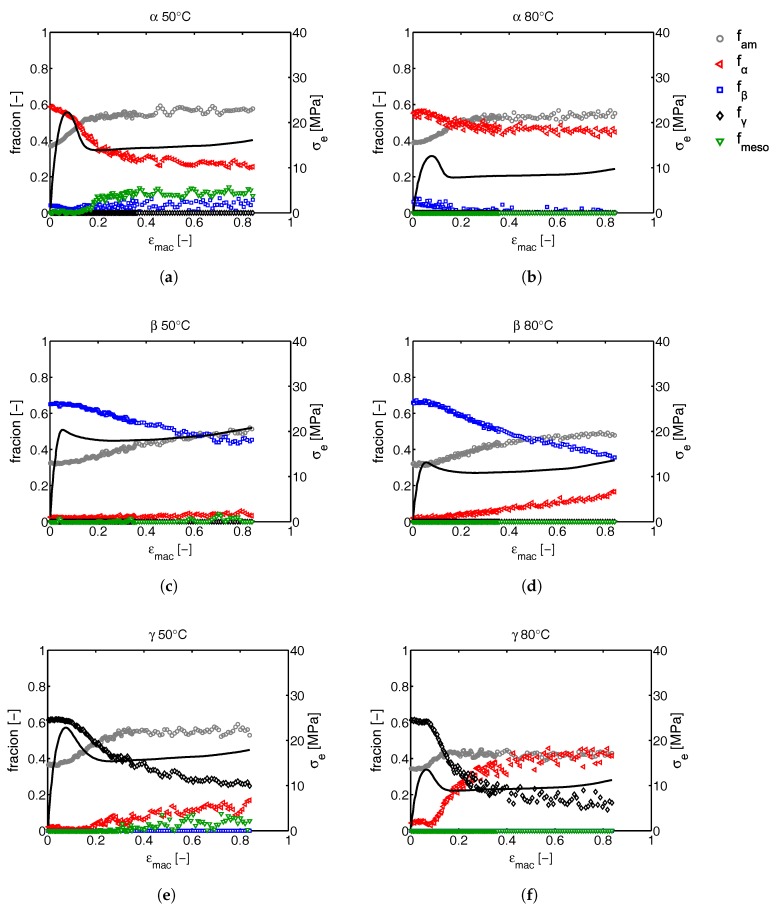
The crystal phase fractions obtained from the WAXD experiments. In (**a**,**c**,**e**), we see the evolution of the crystallinity and phase fractions upon stretching at 50 °C. Figure (**b**,**d**,**f**) show the result of the tensile experiments performed at 80 °C. From top to bottom, we see α, β and γ-iPP.

The results of the approach, discussed in Section 2.3.2, to quantify the crystal phase composition, are given in Figure 18. Here, together with the apparent macroscopic stress-strain response, the phase content of the samples deformed at 25 and 110 °C, is shown as function of the macroscopic strain. Results of tensile experiments performed at 50 and 80 °C can be found in the Appendix Figure A1.

The difference in the structural evolution at room temperature of the α-, β- and γ-samples that immediately draws the attention is the strong and drastic crystal destruction during the macroscopic softening for α- and γ-iPP, which is contrasting to the slow and gradual changes in β-iPP. In α-iPP, almost all the crystals that are initially present in the sample are being destroyed at low temperatures (25 °C), and either transform to oriented mesophase or amorphous phase. At higher temperatures, the amount of newly formed mesophase is lower and ultimately, at a drawing temperature of 110 °C reduces to zero. At the highest temperatures the α-iPP fraction slightly changes, however, based on the results presented in Figure 12 it is known that the crystals partially melt and orient, and that the sample subsequently recrystallizes. The gradual transitions observed upon deformation of β-iPP are also observed at elevated temperatures. Where the transition at low temperatures is mainly from β to amorphous or mesophase, the recrystallization at elevated temperature gives α-iPP. The overall crystallinity slightly decreases at all drawing temperatures. The γ-iPP transforms partially to α-iPP already at a temperature of 50 °C (see the Supporting Information). With increasing temperature the fraction of γ- transforming to α-phase increases, until a temperature of 110 °C where this transition takes place exclusively. To summarize, at low temperatures all polymorphs are being partially destroyed and form amorphous material or oriented mesophase, while at high temperatures all polymorphs transform to oriented α-iPP. In any case the crystallinity slightly decreases. The structural changes observed from X-ray and the coupling to the apparent macroscopic response are affected by geometric effects, rather than pure deformation, due to localization effects. Therefore, in Section 3.2.2, these transitions are investigated by means of uni-axial compression.

**Figure 19:**
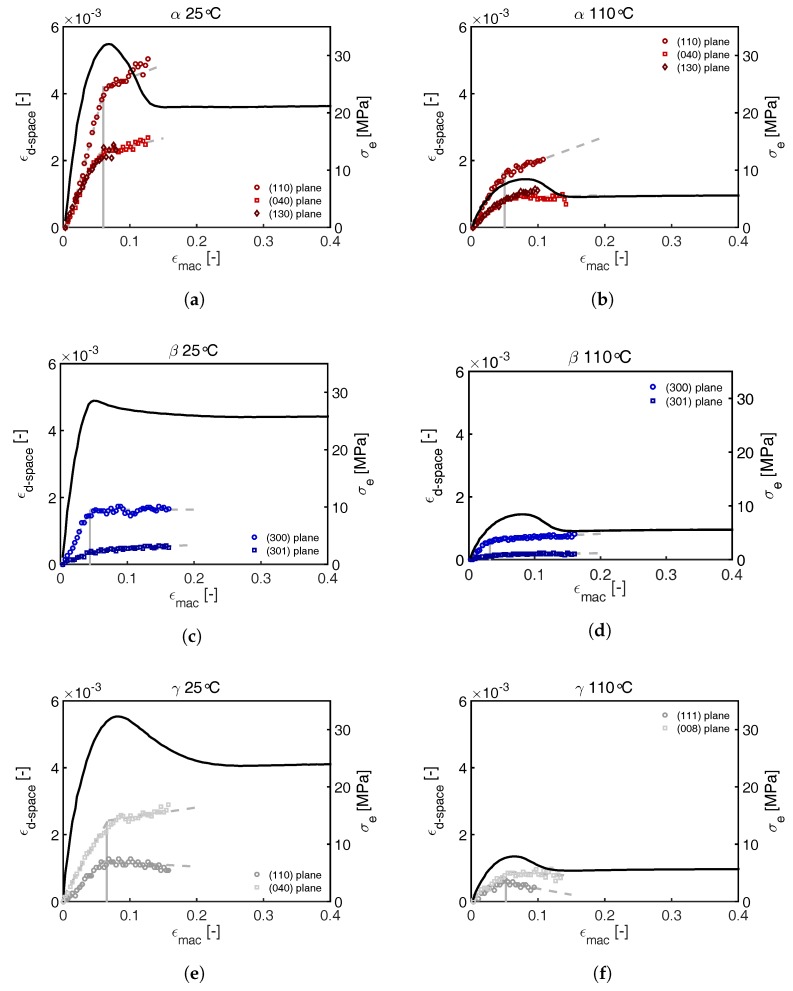
The increase of the distance between crystal planes of (**a**,**b**) α-iPP; (**c**,**d**) β-iPP and (**e**,**f**) γ-iPP. The strain at which the distance between the crystal planes no longer increases following the initial slope is associated with the onset of the plastic deformation. Figure (**a**,**c**,**e**) are obtained from tensile tests performed at 25 °C, while (**b**,**d**,**f**) are taken at 110 °C.

For crystal phase transitions to take place, the chains within the crystal structures need to have a certain amount of mobility, induced by either the applied temperature or stress. From the results presented in Figure 18 it is clear that this mobility is mainly achieved after the yield point. From that point on, the material is able to partially melt and form new oriented structures. The deformation at this stage is plastic. In order to determine the onset of plastic deformation in the crystal, the evolution of the d-spacing is used. The increasing distance between crystal planes in the polar regions of the diffraction pattern is reflected in the d-spacing and thus, the peak positions observed in the WAXD frames. In Figure 19, this evolution in d-spacing is depicted in terms of a strain obtained from Equation (21), and presented as a function of the macroscopically applied strain $\epsilon_{app}$.

As expected for purely elastic behavior, a linear increase in the $\epsilon_{d-space}$ is obtained first. Subsequently this increase levels off, indicating that the further increasing stress, transmitted on the crystals, no longer results in the same increase in d-spacing. This leveling off is clearly observed prior to yielding and can be interpreted as the onset of collective stress-induced α-relaxation. Therefore, the onset of this deviation marks the beginning of crystal plasticity. In the case of β-iPP this transition takes place at low macroscopic strains compared to α- and γ-iPP. The transition correlates to a combination of morphological features like density of the crystal on one hand, and lamellar thickness on the other hand. In α- and γ-iPP, the crystals have a similar thickness (see Section 3.3) and density (Table 1). The crossed stacking of chains in the γ-iPP lamellae seems to slightly postpone plastic deformation as well. Note that prior to yielding this comparison can be made since the deformation is (close to) homogeneous.

#### 3.2.2. Compression Tests (WAXD)

**Figure 20:**
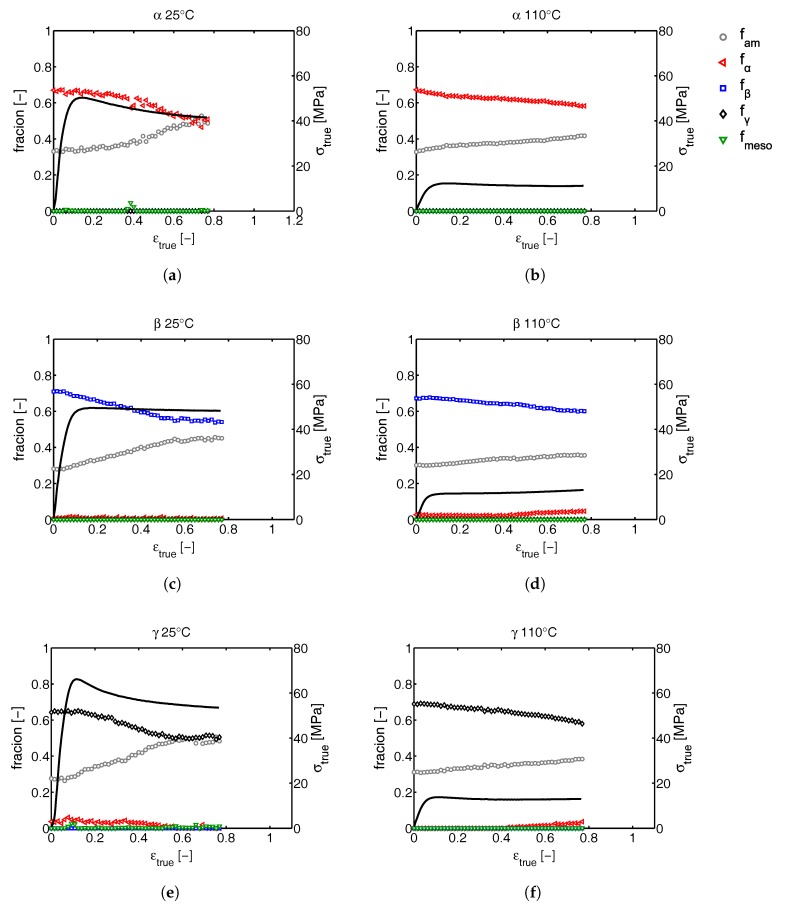
The crystal phase fractions obtained from the WAXD experiments. From top to bottom we see α-, β- and γ-iPP. From left to right we see the evolution of the crystallinity and phase fractions upon compressing at 25 and 110 °C. The true stress as a function of the true strain obtained at a true strain rate of $10^{-2}\;s^{-1}$ is shown with the solid black lines.

Since all the phase transitions are depicted as a function of the macroscopic tensile strain, the question rises how big the effect of localization phenomena are. To answer this question, similar analysis are done on α-, β- and γ-iPP deformed in uni-axial compression experiments at temperatures of 25 and 110 °C, where the applied true strain rate is constant and thus the true stress as a function of the true strain can be obtained, see Figure 20.

The apparent conservation of the structural integrity of β-iPP upon deformation as observed from the tensile experiments (Figure 18) is purely a result of reduced localization compared to α- and γ-iPP. In fact, during compression and at low temperatures it even seems as if the destruction of crystals already starts at relatively low strains, while for α- and γ-iPP the crystallinity starts to decrease mainly after yielding. Furthermore, the compression experiments show no softening in β-iPP, independent of the temperature, whereas in the tensile experiments the β-iPP samples do show softening as a result of geometrical effects. These findings compare well with the observations reported by Xu et al. [57]. Although this work only provides a comparison between α- and β-iPP, the observed phenomena are similar to what is found in this study. α-iPP shows more pronounced strain softening and smaller strain hardening compared to β-iPP. To the knowledge of the authors no publications exist that comment on the strain hardening and softening of γ-iPP in uni-axial compression experiments. The early onset of crystal destruction in β-iPP compared to α-iPP, and the slightly later onset in γ-iPP perfectly matches the findings of Lezak et al., who studied the deformation of α-, β- and γ-iPP in plane strain compression at room temperature [21,25] and at elevated temperature [22,58]. From their extended research they concluded that the initiation of plastic deformation is relatively easy in β-iPP as compared to α-iPP since both crystal slip and shear are easier. For γ-iPP on the other hand, the opposite conclusion was drawn, and plastic deformation was found to be more difficult than in case of α-iPP. This matches with the observations presented on crystal deformation in this work, even though in the tensile experiments the initiation and growth of voids can have a large effect on the local stress states present in the material.

### 3.3. SAXS Analysis

#### 3.3.1. Tensile Tests (SAXS)

**Figure 21:**
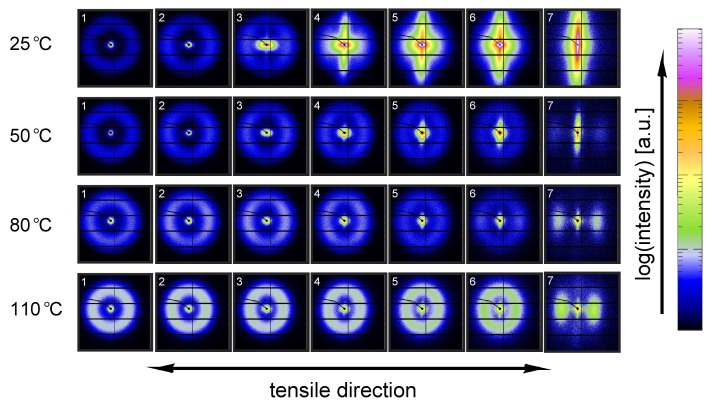
Normalized 2D SAXS patterns of α-iPP stretched at temperatures of 25, 50, 80 and 110 °C from top to bottom. The true strains, determined with the assumption of fully uni-axial deformation, are given as well. The macroscopic strains at which the patterns were taken are indicated by the markers in Figure 8. The stretching direction is horizontal.

To investigate the structural evolution at the nanometer length scale, SAXS data was taken for the same set of experimental conditions as for the WAXD experiments. In Figure 21, the results of the experiments conducted on α-iPP are shown.

The intensity scattered from the lamellae increases with temperature, which can be explained by the difference in thermal expansion between the crystals and the amorphous domains that increases the density contrast. The undeformed samples, with randomly oriented lamellar stacks, give rise to a scattering circle with a homogeneous distribution. Upon deformation, the sample transforms to a state in which the scattering of the lamellae concentrates in the polar regions. At 25 °C voids are already present at the yield point (frame 3), evidenced by the clear lobes near the beam center. From the scattering pattern it becomes clear that the dimensions of the voids initially have the largest dimension in the direction perpendicular to the tensile direction. During strain softening and hardening this gradually evolves into the opposite; the largest dimensions of the voids are now parallel to the stretching direction. This observation is accompanied by a transition from high aspect ratio craze-like features to a highly voided state with shear deformation zones. This transition to micro-necking is related to the entanglement network as was shown by for example Kausch et al. [59] and Ishikawa et al. [34]. At high temperature no clear scattering as a result of voiding is observed at yield, however, close to the center some kind of non-cylindrical pattern is observed. This is an indication for the formation of voids, but with dimensions larger than the detectable length scales. Finally, it is evident that lamellae of the newly formed crystals are strongly oriented with the so-called lamellar normal of the stacks parallel to the tensile direction, meaning that the iPP chains orient along the tensile direction.

**Figure 22:**
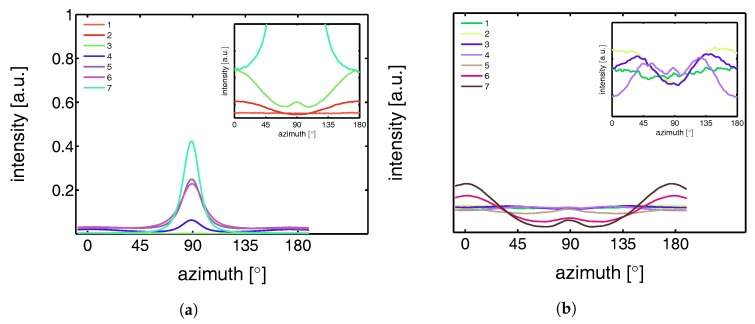
Azimuthal intensity of the lamellar scattering at various strains of α-iPP. (**a**) uni-axial stretching at 25 °C and (**b**) 110 °C. The numbers in the legend correspond to the 2D patterns in Figure 21.

To quantify the lamellar stack orientation upon stretching, azimuthal integrations are performed in a similar way as for the WAXD patterns. In Figure 22 the intensity along the azimuth is shown for the α-sample stretched at room temperature. After yield, in frames 4 to 7, a strong intensity increase in the equatorial regions develops as a result of the transformation of the shape and the growth of fibrillar voids elongated in tensile direction. For this reason, only the first 3 or 4 frames are shown in the inset, i.e., depending on where voiding starts to affect the result. Although the last frame depicted in the insets already shows some features of voiding (development of a peak at 90°), the main intensity distribution during the early stages of deformation is a direct result of the orientation of the lamellar stacks.

An important observation done at 110 °C is that in the initial stages of deformation the intensity of the lamellae concentrates at angles of 45° and 135°. This phenomenon is often attributed to break-up in between lamellae [26,60,61,62], following after cavitation. Here, the scattering patterns show no evidence of voids with a length scale similar to that of the lamellae. Combined with the absence of this preferential orientation in tests performed at room temperature, where there is voiding prior to yielding, it is therefore more likely that the material adopts this orientation because it is preferential for plastic deformation. At room temperature, where we are below $T_{\alpha_{c}}$, a different mechanism takes place which could indicate that the constrained amorphous network is strong enough with respect to the crystallites, to prevent the material to transform to this orientation.

**Figure 23:**
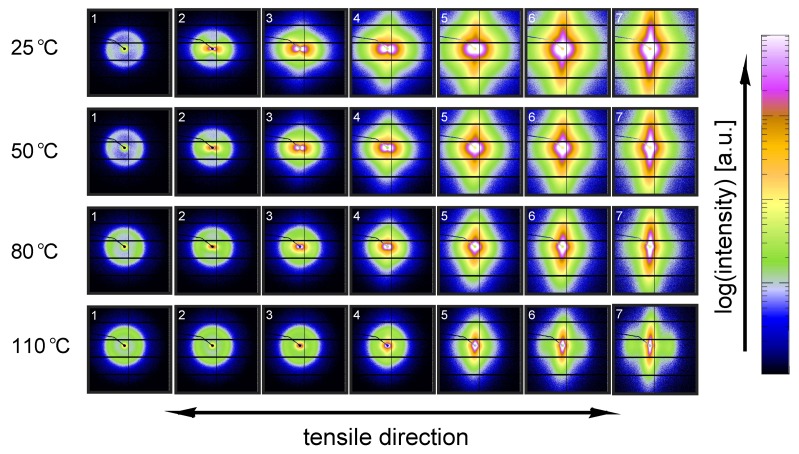
Normalized 2D SAXS patterns of β-iPP stretched at temperatures of 25, 50, 80 and 110 °C from top to bottom. The true strains, determined with the assumption of fully uni-axial deformation, are given as well. The macroscopic strains at which the patterns were taken are indicated by the markers in Figure 8. The stretching direction is horizontal.

The 2D SAXS patterns measured on β-iPP are shown in Figure 23. The scattering intensity close to the center is much stronger than that observed in the α-samples, meaning that the samples exhibit much more voids in the domain of detectable void sizes. This holds for all temperatures. The process of voiding starts already in the early stages of deformation.

The transition from cavities with the largest dimension perpendicular to the tensile direction to cavities with the largest dimension parallel to the tensile direction takes, compared to the α-phase, place at higher strains and seems less clear/unfinished, even in the final stages of the stretching experiment. The scattering from the newly formed crystals appears in the polar regions, indicating that the lamellar normals are oriented parallel to the tensile direction.

**Figure 24:**
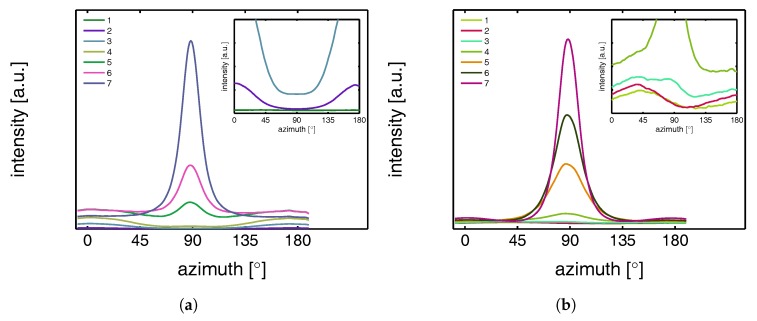
Azimuthal intensity of the lamellar scattering at various strains of β-iPP. (**a**) uni-axial stretching at 25 °C and (**b**) 110 °C. The numbers in the legend correspond to the 2D patterns in Figure 23.

The intensities along the azimuth is shown in Figure 24 upon stretching the sample. As can be observed, the tensile experiment was started from a state which is not completely isotropic. Apparently the compression molding process led to minor orientation in the sample. This only becomes clear after zooming in extensively on the result of an azimuthal integration. Since the anisotropy level is so low, it is assumed that this artifact is not of any influence on the further results. At 25 °C the scattering of the voids affects the integrated intensity almost immediately. At 110 °C cavitation is observed first in the third frame, corresponding to the yield point. Although a small anisotropy seems to be present from the beginning of the experiment there is no evidence for a tendency of the formation of a pattern with a orientation at +45° and −45°, which is different from α-iPP. These observations are similar to the results found by Men et al., who worked on Poly(1-butene) [63], and claimed cavitation through lamellar breakup.

**Figure 25:**
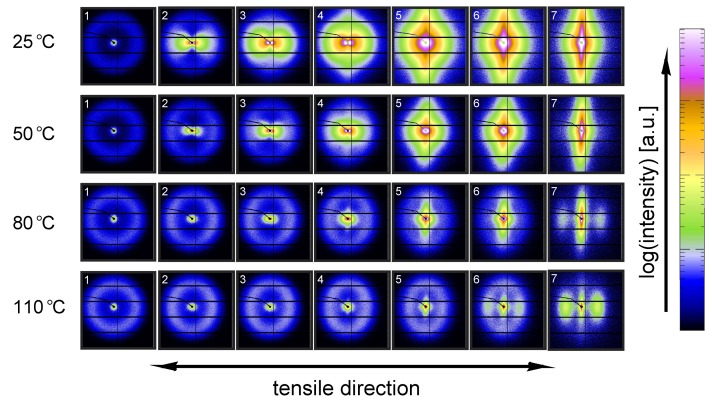
Normalized 2D SAXS patterns of γ-iPP stretched at temperatures of 25, 50, 80 and 110 °C from top to bottom. The true strains, determined with the assumption of fully uni-axial deformation, are given as well. The macroscopic strains at which the patterns were taken are indicated by the markers in Figure 8. The stretching direction is horizontal.

Finally, Figure 25 contains the SAXS patterns taken from tensile experiments performed on γ-iPP. Based on the scattering intensity it is expected that the void fraction (within the detectable range) is in between that of α- and β-iPP. The onset of voiding is clearly before yielding at low temperatures, and also at high temperatures there is some evidence since the scattering close to the center deviates from circular. At 110 °C the scattering of the original lamellae becomes strongly elliptical, before the selective melting and recrystallization into oriented crystallites with the lamellar normal in tensile direction takes place. Similar to the α- and β-iPP the dimensions of the voids are initially larger perpendicular to the tensile direction and transform to shapes that have the largest dimensions parallel to the drawing direction. This happens beyond yielding and takes place more gradual than in case of α-iPP.

**Figure 26:**
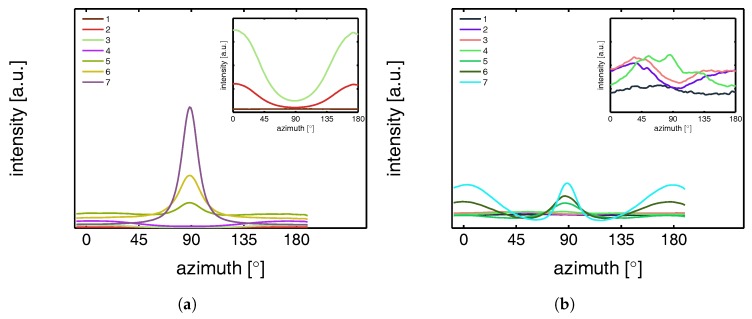
Azimuthal intensity of the lamellar scattering at various strains of γ-iPP. (**a**) uni-axial stretching at 25 °C and (**b**) 110 °C. The numbers in the legend correspond to the 2D patterns in Figure 25.

The intensity as a function of the azimuth is depicted in Figure 26 for the tests performed at 25 and 110 °C. The latter has a preferred orientation at angles of 45° and 135°, which indicates that also in γ-iPP the lamellar break-up or orientation takes place prior to yielding, similar to α-iPP.

**Figure 27:**
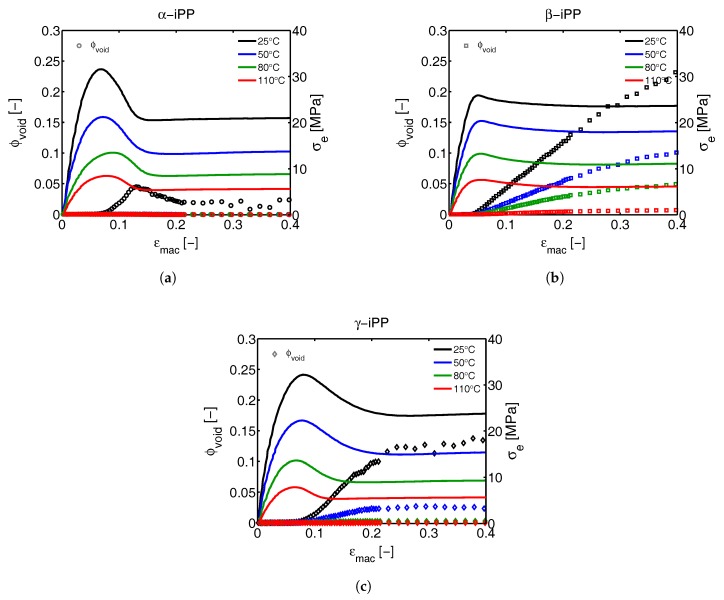
Void volume fraction as a function of the macroscopic strain for α-iPP (**a**), β-iPP (**b**) and γ-iPP (**c**), elongated at 25, 50, 80 and 110 °C.

To quantify the void fraction, the 2D patterns are integrated according to Equation (22), which holds for the assumption of cylindrical symmetry, and by substitution of the result into Equation (23), the evolution of void fraction as a function of the strain is obtained. This procedure was applied on the α-, β- and γ-iPP for all the experimental conditions, and the result is shown in Figure 27. The increase of the void fraction becomes particularly clear at the strain where macroscopic yielding takes place. Based on the 2D images this can be linked to the transition of perpendicularly oriented voids towards voids parallel to the tensile direction, which coincides with a large increase in the scattering intensity. In the case of α-iPP deformed at 25 °C, $\phi_{v}$ increases simultaneously with the macroscopic softening. This increase stops at the end of the softening after which the volume fraction decreases. Since a volume fraction is considered, two possible explanations can be given for this observation: (1) The voids grow (or coalesce) to larger dimensions and therefore are no longer in the detectable size domain; (2) Due to the extension of the voids and the resulting cylindrical dimensions, together with the fibrillar material morphology in between, the negative hydrostatic stresses reduce severely, causing the voids to collapse. This also leads to a reduction in volume.

Quantitative analysis of the void size and shape could confirm the aforementioned hypothesis. To this extent, three different approaches to obtain such information are considered. Unfortunately, due to experimental restrictions none of them turned out to give reliable results. First of all, the approach presented in the work of Lode et al. [64] is considered. This approach was originally used to obtain craze dimensions in amorphous polycarbonate. To successfully fit the parameters of the model of Lode et al., the craze fibril scattering on the equator should show a maximum. Since in the situation at hand, the mean fibrillar spacing is too large, this intensity maximum disappears in the beam stop, making a fit of the parameters highly unreliable. If one wants to use this approach for iPP, USAXS or SALS could possibly be a good alternative for SAXS. Secondly, void dimensions could also be obtained by means of the radius of gyration, as is for example demonstrated by Zafeiropoulos et al. [65], Na et al. [19] or Pawlak et al. [61]. The deformed iPP in this work contains voids of polydisperse dimensions (different populations) and shapes (cylinders, ellipsoids, spheres, etc.). For this reason, an approach similar to Na et al. [19] and Pawlak et al. [61] had to be chosen, in which the total void volume is divided over three populations, each with their own dimension. Unfortunately, the dimensions where again to large to obtain reliable results. Moreover, the void volume does not consist of three discrete void sizes (in practice it is a continuous distribution), making the approach rather sensitive for the arbitrary constraints applied in the optimization routine. Finally, a method introduced by Ruland [66] is considered. This method is successfully used by Lu et al. [20], who obtained dimensions of cavities in iPP. The applicability of this approach depends on the presence of fiber-like entities, which is only the case at very high strains, possibly even higher than the maximum strains reached in this work. Indeed, the application of this method resulted in erroneous void dimensions. In addition to the possible lack of sufficient fiber-like shapes, the setup is aligned such, that the focus is on the sample, rather than on the detector. To summarize, dimensions and shapes of the cavities could not straightforwardly be determined in a reliable way, with either one of the discussed approaches.

When considering the α-iPP elongated at high temperatures, no notable void fraction can be measured although the 2D-patterns clearly show the presence of voids, evidenced by the non-circular scattering close to the beam center. The intensity in these 2D images is given on a logarithmic scale, and thus, the early stage of void initiation immediately becomes clear. Apparently these effects are not strong enough to cause a scattered intensity increase, significantly large to be reflected in the void fraction. In the case of β-iPP the void fraction starts to increase at the yield point and continues to grow throughout the entire experiment. The two phenomena that cause the $\phi_{v}$ in α-iPP to decrease seem not to be present in the β-sample. With increasing temperature, the volume percentage of voids decreases. At 25 and 50 °C, the γ-iPP shows an increase in the scattered intensity that is sufficiently large to be reflected in the void fraction. This starts to develop at the yield point, and continues to grow with increasing strain. However, after softening the void fraction reaches a plateau. The yield stress of β-iPP is found at the lowest strains. Therewith, the onset of voiding in the β samples starts the earliest. This observation is also reported by Pawlak [17]. Although in the work of Pawlak the void volume fraction is not quantified, the 2D SAXS images show that not only the voiding starts at lower macroscopic engineering strain for β-iPP compared to α-iPP, but also that the scattered intensity is much stronger. Since this is directly related to the volume fraction of voids (within the detectable length scale), it can be concluded that the findings in this work are in good agreement with the work of Pawlak. Na et al. [19] investigated the volumetric strain upon stretching α- and γ-iPP at room temperature. At low strains they first found a volumetric increase in γ-iPP, subsequently followed by an increase of the α-iPP volume at slightly higher strains. This seems to disagree with the findings reported here, however, volumetric strain does not necessarily match the void fraction obtained from SAXS experiments in which only part of the void fraction can be assessed, i.e., the part in the detectable length scale. It is very likely that in the case of α- and γ-iPP the void dimensions quickly grow out of the detectable range. Furthermore, determination of the volumetric strain at the early stages of deformation, where nano-scale voids are formed, is a less sensitive technique compared to X-ray scattering. Moreover, crystal phase transitions as demonstrated in the previous sections lead to a change in material density and, therefore, also affect the volumetric strain obtained from optical techniques. To conclude, as far as a good comparison can be made, no insurmountable differences have been found between the results presented in this work and in other studies. In fact, general agreement between the trends observed for different polymorphs is found.

#### 3.3.2. Lamellar Morphology

**Figure 28:**
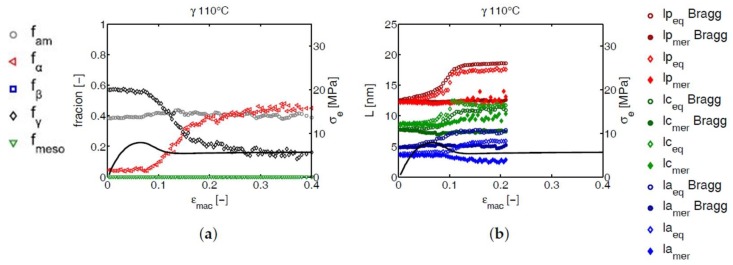
(**a**) Crystal phases in γ-iPP upon stretching at 110 °C; and (**b**), The evolution of the long period, lamellar thickness, and amorphous layer thickness.

**Figure A2:**
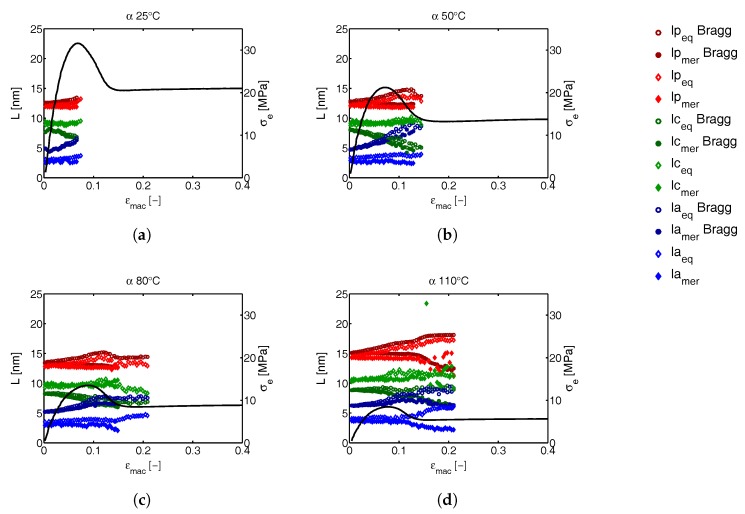
Long period, amorphous layer thickness and lamellar thickness determined via Bragg’s law and the 1D autocorrelation function versus the macroscopic strain for α-iPP, elongated at 25, 50, 80 and 110 °C.

**Figure A3:**
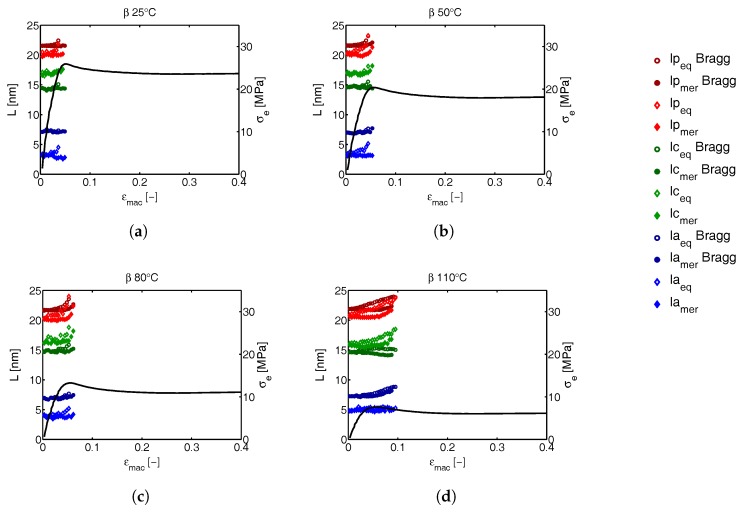
Long period, amorphous layer thickness and lamellar thickness determined via Bragg’s law and the 1D autocorrelation function versus the macroscopic strain for β-iPP, elongated at 25, 50, 80 and 110 °C.

The crystal phase transformations are accompanied by changes in the lamellar morphology. For structural information on the lamellar thickness, the long period and the amorphous layer thickness, in both the tensile and transverse direction, the equatorial and meridional regions were integrated separately, see Figure 6. The integrated 1D intensity was Lorentz corrected and the methods described in Section 2.3.3 were applied. In Figure A2, Figure A3 and Figure A4 of the Supporting Information, the results are presented as a function of the macroscopic strain, in the range where the analysis could be applied, i.e., before crystals were destroyed too much. The most interesting observation was noted during stretching γ-iPP at 110 °C, see Figure 28. As expected, the lamellar thickness obtained using Bragg’s law is lower than the ones obtained by using the auto-correlation function. In the latter case, the dense amorphous transition zones in the vicinity of the crystals add up to the crystal thickness. In that case, self evidently, the amorphous layers are thinner. In the elastic deformation regime, the long period in the equatorial region clearly increases. The evolution of the long period in the meridional region behaves opposite and even displays a decreasing trend. Based on the results obtained from Bragg’s law, it follows that the amorphous regions increase in the initial stages of deformation, however, also the crystalline regions slightly thicken. After yielding, the associated destruction of γ-iPP and the transformation to α-iPP, a sudden increase in long period is observed. This is dominated by the thickening of lamellae, that increase on average from approximately 8–9 nm, to 12–13 nm, see Figure 28. This strong increase is only observed in the γ-samples after yielding at high temperature; see the Supporting Information.

**Figure 29:**
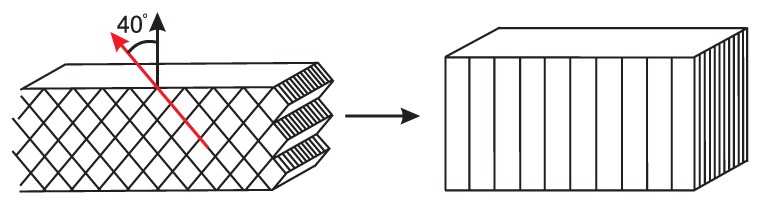
Suggested deformation of a γ-crystal with the orthorhombic unit cell structure at a temperature of 110 °C. The newly formed lamellar crystal is comprised of a monoclinic alpha unit cell structure.

If we consider the γ-crystal lattice and compare it with the newly formed α-lattice, the schematically depicted transformation in Figure 29 is obtained. This transition was investigated in detail, using WAXD experiments, by Auriemma et al. [14], who reported the transformation of γ-iPP (prepared in a low stereo regularity iPP) into α-iPP upon stretching. Their findings and interpretations of the different mechanisms involved at the unit cell level, combined with the increase of the long period simultaneously with the phase transition, suggest that the helical chain conformation is maintained during the transformation. During the destruction of γ-iPP at 110 °C, the ternary helical chain conformation seems to be maintained, and directly incorporated in the newly formed α-lattice. The observation of the increasing lamellar thickness could be worked out further, and offers a special way to obtain α-iPP with large crystal thickness obtained from hot drawing of γ-iPP.

## 4. Conclusions

Tensile and compression experiments were combined with in situ SAXS and WAXD measurements to reveal deformation-induced structural evolution phenomena at multiple length scales for the three well-known polymorphs of iPP. WAXD experiments are used to obtain information about phase transitions, selective melting and orientation of crystal planes. The orientation of lamellae, their thickness and the thickness of the intermediate amorphous layers are derived from the SAXS experiments, as well as the appearance and growth of voids. The findings, with respect to the structural evolution, are linked to the macroscopic intrinsic mechanical response.

Based on our previous work and the intrinsic behavior presented in Section 3.1 and Section 3.2.2, we hypothesize that the typically amorphous phenomenon of softening is mainly due to the constrained amorphous regions in the vicinity of the crystals, rather than deterioration of the crystals.

With respect to the crystallographic structures, the WAXD experiments revealed that at low temperatures, all polymorphs undergo a (partial) phase transition to the oriented mesophase or amorphous phase at large strains. At elevated temperature, the newly formed structure is predominantly the thermodynamically most stable oriented α form. From the compression experiments, it is found that the true strain at which these transitions take place is similar for all polymorphs and, therefore, not the cause for the different macroscopic behavior.

The crystallinity of all polymorphs decreases at similar true strain, independent of the loading conditions, whereas softening is mainly observed in α- and γ-iPP. These are the crystalline forms with the highest density. The extent to which crystals constrain the amorphous phase is dominated by density (secondary interactions) and lamellar thickness or crystal defects. Apparently, the higher density of α and γ-iPP outweighs the influence of thicker lamellae in β-iPP. The crossed configuration of chains in γ-iPP lamellae seems to act as an additional constraint, whereby a rejuvenation treatment is needed in order to obtain the formation and growth of a stable neck in tensile deformation.

To obtain a rejuvenated constrained amorphous phase, mobility has to be created in constrained amorphous layers by either thermal treatments or mechanical deformation. The WAXD experiments show that the β-crystals deform at relatively low strains, clearly prior to yielding. This allows the constrained amorphous domains to gain mobility and soften. Since for β-iPP this happens far before yielding, it might partially explain the absence of softening.

Based on the observation of softening in α- and γ-iPP at a testing temperature of 110 °C, which is above the $\alpha_{c}$-relaxation temperature, it is hypothesized that this is a result of the cross-hatched structure. Break-down of these structures deteriorates the structural integrity, causing a reduction of the stress. In the absence of cross-hatched structures, as is the case for β-iPP, softening is not observed at this temperature.

The evolution of the lamellar orientation suggests that upon stretching, α- and γ-iPP behave differently from β-iPP. In the latter one, the lamellae show no clear orientation while stretching at high temperatures. From the intrinsic behavior, it is found that the strain hardening modulus is the highest in β-iPP. The crystal shear starts the earliest, and also the onset of voiding, which is the most intense in β-iPP, is observed at the lowest strains. The observation that the crystal planes seem to slip before voiding starts suggests that in β-iPP, the critical shear stress is exceeded before the critical cavitation stress is reached.

The γ-iPP, with the crossed chain configuration in the crystal lattice, interestingly shows a strong increase in long period and lamellar thickness while stretching at high temperature. In the softening regime, where the γ-crystals transform to α-crystals, the lamellar thickness in the equatorial regions increases to such an extent that it is plausible to assume that the part of the chain originally incorporated in the crystal maintains the ternary helical conformation during this transition.

The low strain hardening modulus of α-iPP causes the initial disk-like voids to transform into an ellipsoid with a larger shape, oriented in the tensile direction, at relatively low apparent macroscopic strains, compared to the γ- and β-iPP, with the higher strain hardening modulus. In fact, for β- and γ-iPP, this transition is still not completely fulfilled at large macroscopic strains.

Where the crystal structure and topology determine to a large extent the pre-yield behavior and indirectly contribute to the level of the yield stress, the network is decisive in the post-yield behavior (strain hardening). The constrained amorphous phase in the vicinity of the crystalline material is of vital importance for the softening observed after yielding, and the strain hardening modulus determines when voids transform to other shapes.

## Figures and Tables

**Table 1:** List of densities used in Equation (24).

	α	β	γ	meso	amorphous
ρ (kg/m${}^{3}$)	946	921	938	880	850

**Table 2:** Yield stresses obtained in the tensile experiments.

	25 °C	50 °C	80 °C	110 °C
$\sigma_{y}$ of α-iPP (MPa)	32.0	22.3	12.6	8.4
$\sigma_{y}$ of β-iPP (MPa)	28.5	20.4	13.2	7.9
$\sigma_{y}$ of γ-iPP (MPa)	32.3	22.8	13.6	7.9

**Figure A4:**
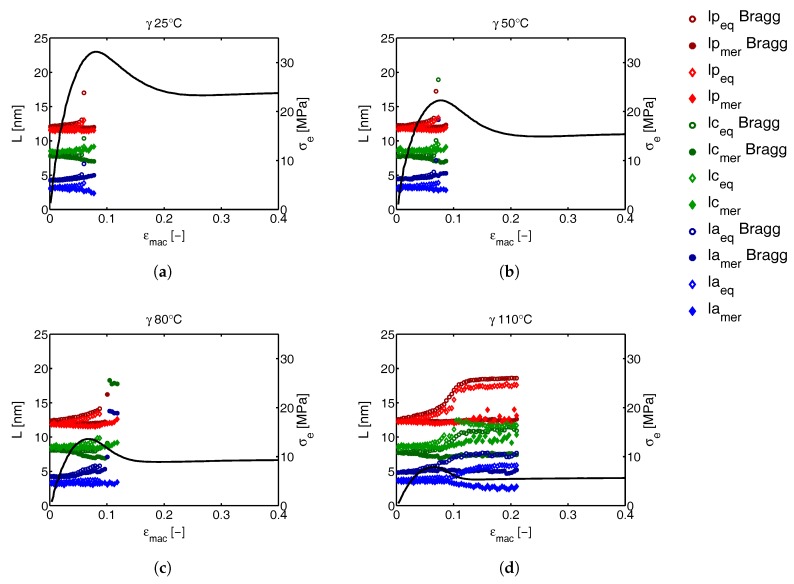
Long period, amorphous layer thickness and lamellar thickness determined via Bragg’s law and the 1D autocorrelation function versus the macroscopic strain for γ-iPP, elongated at 25, 50, 80 and 110 °C.

## Abbreviations

The following abbreviations are used in this manuscript:

iPP	isotactic polypropylene
1D	one-dimensional
2D	two-dimensional
SAXS	small angle X-ray scattering
WAXD	wide angle X-ray diffraction

## References

[1] Brückner S., Meille S., Petraccone V., Pirozzi B. (1991). Polymorphism in isotactic polypropylene. Prog. Polym. Sci..

[2] Lotz B., Wittmann J., Lovinger A. (1996). Structure and morphology of poly(propylenes): A molecular analysis. Polymer.

[3] De Rosa C., Auriemma F., Di Capua A., Resconi L., Guidotti S., Camurati I., Nifant’ev I., Laishevtsev I. (2004). Structure-property correlations in polypropylene from metallocene catalysts: Stereodefective, regioregular isotactic polypropylene. J. Am. Chem. Soc..

[4] Varga J. (2002). *β*-modification of isotactic polypropylene: Preparation, structure, processing, properties, and application. J. Macromol. Sci. Phys..

[5] Roozemond P., Van Erp T., Peters G. (2016). Flow-induced crystallization of isotactic polypropylene: Modeling formation of multiple crystal phases and morphologies. Polymer.

[6] Meille S., Ferro D., Brückner S., Lovinger A., Padden F. (1994). Structure of *β*-isotactic polypropylene: A long-standing structural puzzle. Macromolecules.

[7] Varga J., Karger-Kocsis J. (1996). Rules of supermolecular structure formation in sheared isotactic polypropylene melts. J. Polym. Sci. Part B Polym. Phys..

[8] Varga J., Mudra I., Ehrenstein G. (1999). Highly active thermally stable *β*-nucleating agents for isotactic polypropylene. J. Appl. Polym. Sci..

[9] Valdo Meille S., Brückner S. (1989). Non-parallel chains in crystalline *γ*-isotactic polypropylene. Nature.

[10] Mezghani K., Phillips P. (1998). The *γ*-phase of high molecular weight isotactic polypropylene: III. The equilibrium melting point and the phase diagram. Polymer.

[11] De Rosa C., Auriemma F., Di Girolamo R., De Ballesteros O., Pepe M., Tarallo O., Malafronte A. (2013). Morphology and mechanical properties of the mesomorphic form of isotactic polypropylene in stereodefective polypropylene. Macromolecules.

[12] Caelers H., Parodi E., Cavallo D., Peters G., Govaert L. (2017). Deformation and failure kinetics of iPP polymorphs. J. Polym. Sci. Part B Polym. Phys..

[13] Ma Z., Shao C., Wang X., Zhao B., Li X., An H., Yan T., Li Z., Li L. (2009). Critical stress for drawing-induced *α* crystal-mesophase transition in isotactic polypropylene. Polymer.

[14] Auriemma F., De Rosa C. (2006). Stretching isotactic polypropylene: From “cross-*β*” to crosshatches, from *γ* form to *α* form. Macromolecules.

[15] Zuo F., Keum J., Chen X., Hsiao B., Chen H., Lai S.Y., Wevers R., Li J. (2007). The role of interlamellar chain entanglement in deformation-induced structure changes during uniaxial stretching of isotactic polypropylene. Polymer.

[16] Xiong B., Lame O., Chenal J.M., Rochas C., Seguela R., Vigier G. (2014). In-situ SAXS study of the mesoscale deformation of polyethylene in the pre-yield strain domain: Influence of microstructure and temperature. Polymer.

[17] Pawlak A. (2014). Plastic Deformation and Cavitation in Semi-Crystalline Polymers.

[18] Ran S., Zong X., Fang D., Hsiao B., Chu B., Phillips R. (2001). Structural and morphological studies of isotactic polypropylene fibers during heat/draw deformation by in situ synchrotron SAXS/WAXD. Macromolecules.

[19] Na B., Lv R., Xu W. (2009). Effect of network relaxation on void propagation and failure in isotactic polypropylene at large strain. J. Appl. Polym. Sci..

[20] Lu Y., Wang Y., Chen R., Zhao J., Jiang Z., Men Y. (2015). Cavitation in Isotactic Polypropylene at Large Strains during Tensile Deformation at Elevated Temperatures. Macromolecules.

[21] Lezak E., Bartczak Z., Galeski A. (2006). Plastic deformation behavior of *β*-phase isotactic polypropylene in plane-strain compression at room temperature. Polymer.

[22] Lezak E., Bartczak Z. (2008). Plastic deformation behavior of *β*-isotactic phase isotactic polypropylene in plane-strain compression at elevated temperatures. J. Polym. Sci. Part B Polym. Phys..

[23] Zhang C., Liu G., Song Y., Zhao Y., Wang D. (2015). Structural evolution of *β*-iPP during uniaxial stretching studied by in situ WAXS and SAXS. Polymer.

[24] Jia C., Liao X., Zhu J., An Z., Zhang Q., Yang Q., Li G. (2016). Creep-resistant behavior of *β*-polypropylene with different crystalline morphologies. RSC Adv..

[25] Lezak E., Bartczak Z., Galeski A. (2006). Plastic deformation of the *γ* phase in isotactic polypropylene in plane-strain compression. Macromolecules.

[26] Humbert S., Lame O., Chenal J., Rochas C., Vigier G. (2010). New insight on initiation of cavitation in semicrystalline polymers: In-situ SAXS measurements. Macromolecules.

[27] Wang Y., Jiang Z., Fu L., Lu Y., Men Y. (2014). Lamellar thickness and stretching temperature dependency of cavitation in semicrystalline polymers. PLoS ONE.

[28] Xiong B., Lame O., Chenal J.M., Rochas C., Seguela R., Vigier G. (2015). Temperature-Microstructure Mapping of the Initiation of the Plastic Deformation Processes in Polyethylene via In Situ WAXS and SAXS. Macromolecules.

[29] Xiong B., Lame O., Chenal J., Rochas C., Seguela R., Vigier G. (2013). In-situ SAXS study and modeling of the cavitation/crystal-shear competition in semi-crystalline polymers: Influence of temperature and microstructure in polyethylene. Polymer.

[30] Xiong B., Lame O., Chenal J.M., Rochas C., Seguela R., Vigier G. (2015). Amorphous phase modulus and micro-macro scale relationship in polyethylene via in situ SAXS and WAXS. Macromolecules.

[31] Humbert S., Lame O., Vigier G. (2009). Polyethylene yielding behaviour: What is behind the correlation between yield stress and crystallinity?. Polymer.

[32] Schrauwen B., Janssen R., Govaert L., Meijer H. (2004). Intrinsic deformation behavior of semicrystalline polymers. Macromolecules.

[33] Men Y., Rieger J., Strobl G. (2003). Role of the Entangled Amorphous Network in Tensile Deformation of Semicrystalline Polymers. Phys. Rev. Lett..

[34] Ishikawa M., Ushui K., Kondo Y., Hatada K., Gima S. (1996). Effect of tie molecules on the craze strength of polypropylene. Polymer.

[35] Aboulfaraj M., G’Sell C., Ulrich B., Dahoun A. (1995). In situ observation of the plastic deformation of polypropylene spherulites under uniaxial tension and simple shear in the scanning electron microscope. Polymer.

[36] Labour T., Gauthier C., Séguéla R., Vigier G., Bomal Y., Orange G. (2001). Influence of the *β* crystalline phase on the mechanical properties of unfilled and CaCO3-filled polypropylene. I. Structural and mechanical characterisation. Polymer.

[37] Labour T., Vigier G., Séguéla R., Gauthier C., Orange G., Bomal Y. (2002). Influence of the *β*-crystalline phase on the mechanical properties of unfilled and calcium carbonate-filled polypropylene: Ductile cracking and impact behavior. J. Polym. Sci. Part B Polym. Phys..

[38] Van Drongelen M., Van Erp T., Peters G. (2012). Quantification of non-isothermal, multi-phase crystallization of isotactic polypropylene: The influence of cooling rate and pressure. Polymer.

[39] Haward R. (1993). Strain hardening of thermoplastics. Macromolecules.

[40] Van Melick H., Govaert L., Meijer H. (2003). Localisation phenomena in glassy polymers: Influence of thermal and mechanical history. Polymer.

[41] Bras W., Dolbnya I., Detollenaere D., Van Tol R., Malfois M., Greaves G., Ryan A., Heeley E. (2003). Recent experiments on a combined small-angle/wide-angle X-ray scattering beam line at the ESRF. J. Appl. Crystallogr..

[42] Zhang H., Scholz A., De Crevoisier J., Vion-Loisel F., Besnard G., Hexemer A., Brown H., Kramer E., Creton C. (2012). Nanocavitation in carbon black filled styrene-butadiene rubber under tension detected by real time small angle X-ray scattering. Macromolecules.

[43] Vonk C., Kortleve G. (1967). X-ray small-angle scattering of bulk polyethylene-II. Analyses of the scattering curve. Kolloid Z. Z. Polym..

[44] Ruland W. (1977). The evaluation of the small-angle scattering of lamellar two-phase systems by means of interface distribution functions. Colloid Polym. Sci. Kolloid Z. Z. Polym..

[45] Debye P., Bueche A. (1949). Scattering by an inhomogeneous solid. J. Appl. Phys..

[46] Porod G. (1951). Die Röntgenkleinwinkelstreuung von dichtgepackten kolloiden Systemen-I. Teil. Kolloid Z..

[47] Koberstein J., Morra B., Stein R. (1980). The determination of diffuse-boundary thicknesses of polymers by small-angle X-ray scattering. J. Appl. Crystallogr..

[48] Jansen B., Rastogi S., Meijer H., Lemstra P. (2001). Rubber-modified glassy amorphous polymers prepared via chemically induced phase separation. 2. Mode of microscopic deformation studied by in situ small-angle X-ray scattering during tensile deformation. Macromolecules.

[49] G’Sell C., Jonas J. (1981). Yield and transient effects during the plastic deformation of solid polymers. J. Mater. Sci..

[50] Smit R., Brekelmans W., Meijer H. (1999). Prediction of the large-strain mechanical response of heterogeneous polymer systems: Local and global deformation behaviour of a representative volume element of voided polycarbonate. J. Mech. Phys. Solids.

[51] Caelers H., Govaert L., Peters G. (2016). The prediction of mechanical performance of isotactic polypropylene on the basis of processing conditions. Polymer.

[52] Meijer H., Govaert L. (2005). Mechanical performance of polymer systems: The relation between structure and properties. Prog. Polym. Sci..

[53] Mezghani K., Phillips P. (1997). The *γ*-phase of high molecular weight isotactic polypropylene. II: The morphology of the *γ*-form crystallized at 200 MPa. Polymer.

[54] Yamada K., Matsumoto S., Tagashira K., Hikosaka M. (1998). Isotacticity dependence of spherulitic morphology of isotactic polypropylene. Polymer.

[55] Von Compostella M., Coen A., Bertinotti F. (1962). Fasern und Filme aus isotaktischem Polypropylen. Angew. Chem..

[56] Lotz B., Wittmann J. (1986). The molecular origin of lamellar branching in the *α* (monoclinic) form of isotactic polypropylene. J. Polym. Sci. Part B Polym. Phys..

[57] Xu W., Martin D., Arruda E. (2005). Finite strain response, microstructural evolution and *β* → *α* phase transformation of crystalline isotactic polypropylene. Polymer.

[58] Lezak E., Bartczak Z. (2007). Plastic deformation of the *γ* phase isotactic polypropylene in plane-strain compression at elevated temperatures. Macromolecules.

[59] Plummer C., Kausch H.H. (1996). Micronecking in thin films of isotactic polypropylene. Macromol. Chem. Phys..

[60] Butler M., Donald A., Ryan A. (1998). Time resolved simultaneous small- and wide-angle X-ray scattering during polyethylene deformation-II. Cold drawing of linear polyethylene. Polymer.

[61] Pawlak A. (2007). Cavitation during tensile deformation of high-density polyethylene. Polymer.

[62] Pawlak A., Galeski A. (2005). Plastic deformation of crystalline polymers: The role of cavitation and crystal plasticity. Macromolecules.

[63] Men Y., Rieger J., Homeyer J. (2004). Synchrotron ultrasmall-angle X-ray scattering studies on tensile deformation of poly(1-butene). Macromolecules.

[64] Lode U., Pomper T., Karl A., Von Krosigk G., Cunis S., Wilke W., Gehrke R. (1998). Development of crazes in polycarbonate, investigated by ultra small angle X-ray scattering of synchrotron radiation. Macromol. Rapid Commun..

[65] Zafeiropoulos N., Davies R., Roth S., Burghammer M., Schneider K., Riekel C., Stamm M. (2005). Microfocus X-ray scattering scanning microscopy for polymer applications. Macromol. Rapid Commun..

[66] Ruland W. (1969). Small- angle scattering studies on carbonized cellulose fibers. J. Polym. Sci. Polym. Symp..

